# A plasma membrane Ca^2+^-dependent protein kinase PtCDPK2 promotes phosphorus starvation resilience in *Phaeodactylum tricornutum*

**DOI:** 10.1093/jxb/erag114

**Published:** 2026-02-28

**Authors:** Yasmin Meeda, Ellen L Harrison, Susan Wharam, Heather O’Keefe, Andrea Highfield, Adam Monier, Glen L Wheeler, Katherine E Helliwell

**Affiliations:** Marine Biological Association, Citadel Hill, Plymouth PL1 2PB, UK; Biosciences, University of Exeter, Exeter EX4 4QD, UK; Marine Biological Association, Citadel Hill, Plymouth PL1 2PB, UK; Biosciences, University of Exeter, Exeter EX4 4QD, UK; Marine Biological Association, Citadel Hill, Plymouth PL1 2PB, UK; Marine Biological Association, Citadel Hill, Plymouth PL1 2PB, UK; Marine Biological Association, Citadel Hill, Plymouth PL1 2PB, UK; Living Systems Institute, University of Exeter, Exeter EX4 4QD, UK; Marine Biological Association, Citadel Hill, Plymouth PL1 2PB, UK; Marine Biological Association, Citadel Hill, Plymouth PL1 2PB, UK; Biosciences, University of Exeter, Exeter EX4 4QD, UK; Lawrence Berkeley National Laboratory, USA

**Keywords:** Ca^2+^ signalling, calcium, CDPKs, CRISPR/Cas9, diatoms, kinases, nutrient signalling, *Phaeodactylum*, phosphorus

## Abstract

Phosphorus (P) is an essential element limiting algal growth in aquatic ecosystems. Diatoms are abundant microalgae that thrive in nutrient-variable environments. Determining how diatoms regulate responses to P availability is thus crucial for understanding their ecological success. P-limited diatoms use Ca^2+^-dependent signalling to sense and coordinate responses to phosphate resupply. However, the apparatus enabling Ca^2+^ signal decoding in diatoms remains poorly understood. *Phaeodactylum tricornutum* possesses several Ca^2+^-dependent protein kinases (CDPKs) that are up-regulated by P starvation, although it is unknown whether they act to coordinate P starvation responses and/or transduce Ca^2+^ signals stimulated by P resupply. Here, we functionally characterized *PtCDPK2*. We show that PtCDPK2 localizes to the cell periphery, suggesting a role regulating plasma membrane processes. PtCDPK2 is co-expressed with the P starvation response regulator, PtPSR1. Increases in PtCDPK2 are also coordinated with the capacity for P–Ca^2+^ signalling. Quantification of extracellular phosphate indicates that the activation of P–Ca^2+^ signalling ability under P starvation is not controlled by external phosphate exhaustion, but decreased cellular P quota. Finally, *Ptcdpk2* mutants have significantly reduced *F*_v_/*F*_m_ and alkaline phosphatase activity under P starvation, indicating that they are impaired in their ability to cope with P depletion. Together, our findings demonstrate that Ca^2+^ signalling processes contribute to diatom tolerance to P limitation, alongside their established role mediating P resupply responses.

## Teaser

Characterization of a Ca^2+^-dependent kinase (CDPK) in the model diatom *Phaeodactylum tricornutum* reveals a role for PtCDPK2 in coping with P limitation, and establishes a foundation for understanding Ca^2+^ signal decoding in diatoms.

## Introduction

Phosphorus (P) is a crucial macronutrient sustaining life in aquatic environments. P has vital roles as a constituent of key molecules including nucleic acids, phospholipids, and ATP, as well as mediating cell signalling (Bünemann and Condron, 2007; Paytan and McLaughlin, 2007). P limits algal growth in lakes, and coastal as well as open ocean ecosystems (Thingstad *et al*., 1998; Hudson *et al*., 2000; Ly *et al*., 2014). Moreover, changing anthropogenic nutrient inputs are exacerbating P limitation in coastal habitats (Seitzinger *et al*., 2010; Burson *et al*., 2016). P availability can fluctuate considerably due to river run-off, atmospheric deposition, and nutrient up-welling (Jordan and Joint, 1998; Karl, 2014; Duhamel, 2025). Whilst inorganic orthophosphate (PO_4_^3−^) is the preferred form of P utilized by plants and algae, dissolved organic P (DOP) often exceeds phosphate concentrations in marine ecosystems (Duhamel *et al*., 2021), sustaining up to 75% of annual net community production (Letscher *et al*., 2022). Mechanisms enabling algae to sense and respond to varying P availability, and access organic P pools, thus govern their competitive success in aquatic environments.

Diatoms (stramenopiles) contribute ∼40% of marine primary productivity (Armbrust, 2009; Malviya *et al*., 2016), and are emerging as powerful model systems for algal cell biology (Armbrust *et al*., 2004; Bowler *et al*., 2008; Nymark *et al*., 2016; Mock *et al*., 2017; Falciatore *et al*., 2020; Villar *et al*., 2025). Genomic and genetic resources have yielded important insights into how diatoms cope with P starvation. Diatoms induce phosphate transporters and alkaline phosphatases to maximize P scavenging and acquisition (Dyhrman *et al*., 2012; Lin *et al*., 2013; Cruz de Carvalho *et al*., 2016; Alipanah *et al*., 2018; Matsui *et al*., 2024). Intracellular stores of polyphosphate (poly-P) are also consumed under P deficiency (Lapointe *et al*., 2024), and cellular P usage is economized, for instance via substitution of phospholipids (e.g. with sulfolipids) (Van Mooy *et al*., 2009; Martin *et al*., 2011). P limitation also causes reductions in nitrogen (N) uptake and photosynthetic efficiency (Cruz de Carvalho *et al*., 2016; Helliwell *et al*., 2021a; You *et al*., 2022).

Despite belonging to a different eukaryotic supergroup compared with green algae and plants (Dorrell and Smith, 2011), diatoms share key P starvation regulatory machinery. The MYB-like transcription factor phosphate starvation regulator 1 (PSR1), first identified in *Chlamydomonas reinhardtii* (Wykoff *et al*., 1999) and subsequently in *Arabidopsis thaliana* (known as PHR1) (Rubio *et al*., 2001), is conserved in diverse algae including diatoms (Ngan *et al*., 2015; Sharma *et al*., 2020; Fiore *et al*., 2021; Helliwell, 2023). P-starved *C. reinhardtii Crpsr1* mutants have differential gene expression compared with the wild type (WT) (Moseley *et al*., 2006). However, overlap between P-responsive target genes of PSR1/PHR1 is limited between *C. reinhardtii* and *A. thaliana* (Hammond *et al*., 2003; Wu *et al*., 2003; Moseley *et al*., 2006). In the model pennate diatom *Phaeodactylum tricornutum*, *PtPSR1* is also necessary for regulating P starvation responses, with *Ptpsr1* mutants showing decreased induction of alkaline phosphatase activity and phospholipid degradation (Sharma *et al*., 2020). However, differences in P signalling mechanisms are apparent between photosynthetic eukaryotes. For instance, P-starved diatoms *P. tricornutum* and *Thalassiosira pseudonana* sense phosphate resupply via Ca^2+^ signalling (Helliwell *et al*., 2021a), unlike *C. reinhardtii* and plants (*A. thaliana*) (Matthus *et al*., 2019; Pivato *et al*., 2025). Inhibition of P–Ca^2+^ signals using the inhibitor ruthenium red (RuR) reduces the ability of cells to mediate rapid enhancements in N uptake necessary for recovering from P deficiency (Helliwell *et al*., 2021a). Moreover, this P–Ca^2+^ signalling response is impaired in *Ptpsr1* mutants (Harrison *et al*., 2025), suggesting direct or indirect regulation of P–Ca^2+^ signalling machinery by PtPSR1 and demonstrating interplay between P starvation and sensing mechanisms. Nevertheless, the molecular machinery underlying P–Ca^2+^ signalling, including how signals are generated and decoded, remains unknown.

Like other eukaryotes, diatoms use Ca^2+^ signalling to perceive an array of stimuli including mechanical stress, nutrients (phosphate and iron), allelochemicals, temperature, light, membrane depolarization, and osmotic shock (Falciatore *et al*., 2000; Vardi *et al*., 2006; Helliwell *et al*., 2019, 2021b; Kleiner *et al*., 2022; Flori *et al*., 2024). *In silico* studies have revealed components of the diatom Ca^2+^ signalling toolkit, including the ion channel repertoire of different species (Verret *et al*., 2010; Murphy *et al*., 2024). Moreover, functional studies have enabled characterization of a novel single-domain voltage-gated Ca^2+^ channel (Helliwell *et al*., 2019) and Ca^2+^ efflux proteins (Liu *et al*., 2024) in diatoms. However, the diatom Ca^2+^ sensor apparatus has received little attention. Ca^2+^ sensor kinases can detect specific Ca^2+^ signals (or signatures) arising from external stimuli, to induce downstream responses via phosphorylation (Harper *et al*., 2004; Dodd *et al*., 2010). Eukaryotic genomes encode distinct families of Ca^2+^ sensors (Bredow and Monaghan, 2019). For instance, multicellular plants have evolved an expanded repertoire of calcineurin B-like (CBL)-interacting protein kinases (CIPKs) and Ca^2+^-dependent protein kinases (CDPKs) (e.g. 10 and 26 CBL/CIPKs, respectively, and 34 CDPKs in *A. thaliana*) (Edel *et al*., 2017). Of particular note are the CDPKs that combine Ca^2+^ sensing and transduction capabilities, via a calmodulin-like domain typically with four Ca^2+^-binding EF-hands, fused to a C-terminal Ser/Thr kinase domain (Harper *et al*., 1991; Wernimont *et al*., 2010). These proteins play crucial roles in plant nutrient sensing, including transducing nitrate-induced Ca^2+^ signals in *A. thaliana* to control primary nitrate responses (Riveras *et al*., 2015; Liu *et al*., 2017, 2020).

Whereas CBL/CIPKs are absent in diatoms (Beckmann *et al*., 2016), *P. tricornutum* elevates transcriptional expression of several *CDPK* genes under P limitation that contain PtPSR1 motifs in their promoter regions (Sharma *et al*., 2020). This suggests that CDPKs may be required to detect P resupply-induced Ca^2+^ elevations, or coordinate acclimation to P limitation. To investigate these scenarios, we examined *P. tricornutum* CDPKs, including localization, expression, and functional roles of PtCDPK2 in response to P limitation and resupply, and in relation to P–Ca^2+^ signalling and PtPSR1. Additionally, examining the distribution of CDPKs in diatoms more broadly, we identify expansions of the CDPK repertoire in many diatom species.

## Materials and methods

### Strains and culturing

The background strain used in this study was *P. tricornutum* CCAP 1055/1. This strain was used to generate the PtCDPK2–mVenus lines, as well as the *Ptcdpk2* mutants and their subsequent derivative lines expressing the genetically encoded Ca^2+^ biosensor R-GECO1–mTurquoise (RGMT) (Harrison *et al*., 2025). Additionally, certain Ca^2+^ signalling experiments as indicated in the text, were conducted with a genetically modified strain of *P. tricornutum* CCAP 1055/1 (PtR1) expressing R-GECO1 (Helliwell *et al*., 2019). The PtPSR1–mVenus line was described in Harrison *et al*. (2025). Cultures were grown in filtered seawater (FSW) with f/2 nutrients -Si (Guillard and Ryther, 1962) at 18 °C under a 16 h:18 h light:dark cycle with a light intensity of 60–80 µmol m^−2^ s, unless otherwise stated.

All physiology experiments were inoculated to a starting density of 3×10^4^ cells ml^−1^ into high (36 µM initial phosphate) or low (1.8 µM initial phosphate) phosphate, or phosphate resupply (1.8 µM phosphate-grown cells resupplied with 36 µM phosphate on day 4) treatments. Cell counts of *P. tricornutum* were performed at the same time each day using a Beckman Coulter Counter (Beckman Coulter, UK Ltd, Buckinghamshire, UK) with a 3–9 µm aperture. Growth rates were calculated using the equation: μ&ln(*N*2/*N*1)/(*t*2−*t*1), where μ is the specific growth rate, and N1 and N2 are the cell counts at time 1 (*t*1) and time 2 (*t*2), respectively.

### Photosynthetic efficiency measurements

Photosynthetic efficiency (*F*_v_/*F*_m_; maximum quantum yield of PSII) was measured in triplicate with cells dark-adapted for 20 min prior to analysis. Measurements were taken using the PAM fluorometer (Water-PAM, Walz, Germany) with 3 ml cuvettes. The gain was set at 5 for all measurements.

### Identification of *PtCDPK* genes in *P. tricornutum*

To identify CDPKs in the *P. tricornutum* CCAP 1055/1 genome (v3) (Bowler *et al*., 2008; Villar *et al*., 2025), an initial sequence similarity search using *A. thaliana* CPK1 (Uniprot Q06850) (Harper *et al*., 1993, 1994) as a query sequence was performed using BLASTp against the DiatomicBase v3 genome portal using default parameters (Villar *et al*., 2025). The *P. tricornutum* PtCDPK2 (protein ID 21006) was used subsequently to further examine the presence of CDPKs in the *P. tricornutum* genome. Nine CDPK-like hits containing both EF-hand (IPR002048) domain(s) and a kinase (IPR000719) domain were verified using Interpro analysis (Apweiler *et al*., 2000).

### Identification of *CDPK* genes from genome and transcriptome databases

Diatom genomes and transcriptomes were searched using PtCDPK2 as a query, with an E-value cut-off score of 1E^−25^. The *T. pseudonana* (Armbrust, 2009), *Thalassiosira oceanica* (Lommer *et al*., 2012), and *Fragilariopsis cylindrus* (Mock *et al*., 2017) genomes were obtained from the Joint Genome Institute (JGI). The genome of *Fistulifera solaris* (Maeda *et al*., 2022) was searched on UniProt. The transcriptome databases were obtained from the Marine Microbial Eukaryote Sequencing Project (MMETSP) (Keeling *et al*., 2014). Databases were added to Geneious Prime (version 2020.1.1), and hits analysed to determine at least one kinase and one EF-hand domain using the Interpro Plug-in. Duplicates were identified using MAFFT sequence alignment (Katoh *et al*., 2002), compared in MEGAX (version 10.1.8) (Kumar *et al*., 2016), and then removed from further investigation.

### Phylogenetic analysis

For the reconstruction of the phylogenetic tree, protein sequences from *C. reinhardtii* were obtained from Li *et al*. (2019), and plant sequences were taken from Xiao *et al*. (2016), Yip Delormel and Boudsocq (2019), and Bredow and Monaghan (2022). In addition to *P. tricornutum* CDPKs, hits from the diatoms *Pseudo-nitzschia multiseries* CLN-47 (genome v1) and *T. pseudonana* CCMP 1335 (genome v3) were also included, searched via the JGI portal. Protein sequences were aligned using MAFFT v7 webserver, utilizing default parameters (Kuraku *et al*., 2013; Katoh *et al*., 2019). The final alignment was 464 amino acids. MEGAX software (Kumar *et al*., 2018) was employed to identify the best-fitting evolutionary model using the corrected Akaike information criterion, and a maximum likelihood tree was generated using the WAG+F model with 100 non-parametric bootstrap replicates.

### PtCDPK2–mVenus construct design and strain generation

To generate the PtCDPK2–mVenus construct, a 2604 bp sequence (including flanking restriction sites) for gene ID 21006 was synthezised and custom cloned by Genscript (GenScript, Piscataway, NJ, USA), via *Pst*I and *Stu*I, into the pPha-T1-mVenus vector [a derivative of the pPHA-T1 (accession AF219942) adapted as described by Helliwell *et al*. (2019) containing a codon-optimized mVenus gene (AJN91098.1)]. The synthesized sequence included the start codon (ATG) as well as a 700 bp upstream promoter region, alongside the gene sequence including exons and introns, but excluding the stop codon (Supplementary Fig. S1). The resulting plasmid was transformed into *P. tricornutum* via biolistic transformation with zeocin selection (75 µg ml^–1^) (Helliwell *et al*., 2019). Transformants were screened for mVenus fluorescence using the Leica SP8 confocal microscope with a ×63 oil objective. Cells were excited with a 488 nm argon/krypton laser and emission was detected between 500 nm and 530 nm for mVenus and between 650 nm and 710 nm for chlorophyll autofluorescence. For FM4-64 staining, PtCDPK2–mVenus cells were grown in f/2 medium -Si for 4 d, before addition of 10 μM FM4-64 and incubation for 20 min at room temperature. Cells were visualized with a 488 nm laser, and the emission signals were collected by detection windows between 606 nm and 650 nm on a Leica SP8 confocal microscope.

### Measurement of mVenus reporter lines grown in different P regimes

PtCDPK2–mVenus, PtPSR1–mVenus, and WT *P. tricornutum* lines were inoculated into 24-well plates (catalogue no. 83.3922, SARSTEDT) in a 2 ml volume and grown for 4 d in high (36 µM phosphate), low (1.8 µM phosphate), or resupply (i.e. 4-day-old low-P-grown cells supplemented with 36 µM phosphate) conditions. Chlorophyll fluorescence was measured daily using a CLARIOstar Plus plate reader (BMG Labtech) with 440±9/680±20 nm excitation/emission wavelengths. mVenus fluorescence was measured using 515±10 nm/550±10 nm excitation/emission wavelengths, and was normalized to chlorophyll fluorescence to calculate relative fluorescence values.

### Ca^2+^ imaging experiments to define thresholds of P depletion to activate the P–Ca^2+^ signalling response

*Phaeodactylum tricornutum* strain PtR1 (Helliwell *et al*., 2021a) was grown in artificial seawater (ASW) using Tropic Marin Classic (Tropic Marin®) sea salts (30 g l^–1^) with pH adjusted to 8.1 and sterile filtered with f/2 nutrients -Si, with phosphate provided as indicated in the Results. Glass-bottomed dishes 35 mm in diameter (In Vitro Scientific, Sunnyvale, CA, USA) were coated with 0.01% poly-L-lysine (Sigma-Aldrich, USA) and washed with 1 ml of ASW without nutrients, and PtR1 cells (on day 4 and day 5) were added and left for 10–30 min to settle. Cells were imaged at 20 °C using a Leica DMi8 microscope (Leica, Germany) ×63 oil immersion objective. R-GECO1 cells were excited with 530–555 nm excitation, and emission was measured at 575–630 nm, using a SpectraX LED light source (Lumencor). Image capture and analysis were carried out on Leica Application Suite X. Cells were perfused with ASW for 30 s using a gravity-fed perfusion system, then, for phosphate resupply treatments, cells were perfused with ASW with 36 μM phosphate (NaH_2_PO_4_) for 30 s, unless stated otherwise.

### Generation of *P. tricornutum Ptcdpk2* mutants

The CRISPR/Cas9 [clustered regularly interspaced palindromic repeats (CRISPR)/CRISPR-associated protein 9] vector developed by Nymark *et al*. (2016) was employed for editing the *PtCDPK2* gene (protein ID 21006) in *P. tricornutum* (Nymark *et al*., 2016). We designed two single guide RNAs (sgRNAs) targeted to generate an ∼342 bp deletion. A library of candidate sgRNAs was generated using PHYTOCRISPEX (Rastogi *et al*., 2016) with default parameters (NGG PAM, and CRISPR start from ‘G’). The Broad Institute sgRNA design program (Doench *et al*., 2016; Sanson *et al*., 2018) was used to obtain ‘on-target’ efficiency scores. Two 20 bp guide RNAs (sgRNA1_Pt21006: GAAACAGAACAACAAAAGGA and sgRNA2_Pt21006: GAAGTATTGGAGTTGTGTGA) that passed the PHYTOCRISPEX OFF-target criteria were chosen based on their ON-target scores (0.57 and 0.66, respectively) and position within the gene. Target sgRNAs were predicted to disrupt the kinase domain of the protein. A 483 bp DNA fragment was synthesized (Genscript) containing sgRNA1_Pt21006 and sgRNA2_Pt21006, respectively (Supplementary Fig. S2) (Hopes *et al*., 2016). Additionally, a U6 promoter region was included in the cassette to drive expression of sgRNA2_Pt21006, as well as *Bsa*I restriction site overhangs to clone the DNA fragment into the pKSdiaCas9_sgRNA (Addgene: 74923) plasmid. Note, a second U6 promoter is present in the pKSdiaCas9_sgRNA plasmid that is positioned upstream of sgRNA1_Pt21006, upon cloning of the guide RNA cassette into pKSdiaCas9_sgRNA via *Bsa*I. The resulting plasmid was co-transformed along with pPHAT1 (accession no. AF219942) conferring zeocin resistance using biolistic transformation and 75 µg ml^–1^ zeocin for selection (Helliwell *et al*., 2019). Putative *Ptcdpk2* mutant colonies were screened via PCR using the Phire Plant Direct PCR Kit (ThermoFisher Scientific) with primers targeting the *PtCDPK2* gene: KEH_409F: CGTCCAAGTGCTCGGTAATG and KEH_410R: GTCTCATCTGGCAAGCGTTC. Mutants were further purified by diluting primary colonies suspended in FSW and plating on selection plates (50% FSW f/2 with 75 µg ml^–1^ zeocin). Resulting colonies were picked and re-screened via PCR. DNA sequencing produced clean (unmixed) chromatograms for both *Ptcdpk2.1* and *Ptcdpk2.4*, with sequencing information provided in Supplementary Figs S3 and S4. Coding sequences of the mutant and WT were further analysed via reverse transcription–PCR (RT–PCR).

### RNA extraction and RT–PCR of the *PtCDPK2* gene in *Ptcdpk2* mutants

To further clarify the resulting coding sequences of the WT and *Ptcdpk2* mutants, RT–PCR was performed. Strains were grown in 100 ml of f/2 medium with 1.8 µM phosphate, without silicate and vitamins (to an OD_730_ of ∼0.1, and 1–2×10^6^ cells ml^–1^). Cells were harvested in 50 ml aliquots by centrifugation (4000 *g*, 10 min). Cell pellets were weighed then snap-frozen in liquid nitrogen for storage at −80 °C prior to processing. RNA was extracted from cell pellets (≤100 mg wet weight) using the RNeasy Plant Mini Kit (Qiagen). Cells were thawed on ice in 450 µl of RLT (a lysis buffer containing guanidine isothiocycanate, Qiagen RNeasy kit), with additional 1% v/v β-mercaptoethanol and ∼300 µl (equivalent volume) of glass beads (0.1–1.5 mm, autoclaved then pre-soaked in RLT buffer overnight). Cells were lysed by vortexing (5×10 s, alternated with 10 s chilling on ice). Cell debris and unlysed cells were removed by centrifugation (200 *g*, 10 s, then 17 000 *g*, for 1 min). RNA was processed according to the manufacturer’s instructions, except that the column was washed three times with RPE buffer. DNA contamination was eliminated using a Turbo DNA-*free* Kit (Invitrogen, as in the manufacturer’s instructions) and RNA was quantified by NanoDrop (Thermo Scientific) and frozen in aliquots at −80 °C.

RT–PCR was performed on the RNA samples (∼20 ng) using a One*Taq* One-Step RT–PCR kit (NEB, according to manufacturer’s instructions), with primers keh409F CGTCCAAGTGCTCGGTAATG and keh410R GTCTCATCTGGCAAGCGTTC. RT–PCR cycling conditions were: 48 °C for 15 min (cDNA synthesis step), followed immediately by 94 °C 60 s, 40× (94 °C 15 s, 53 °C 30 s, 68 °C 60 s), and 68 °C 5 min. Negative controls, using the same primers but PCR rather than RT–PCR conditions (standard *Taq* instead of the One*Taq* RT–PCR enzyme mix, and no initial 15 min at 48 °C), were conducted to confirm lack of DNA contamination of RNA samples. As a further control, purified genomic DNA was amplified by these PCR conditions. RT–PCR products and PCR controls were analysed by gel electrophoresis (1% agarose, stained with GelRed), and sequenced (Source BioScience, via Sanger sequencing with primers keh409F and keh410R).

For mutant *Ptcdpk2.4*, PCR amplification of genomic DNA produced a single band, with sequencing indicating a pure mutant. However, RT–PCR yielded a double band despite no evidence of DNA contamination in the no-reverse transcription controls, and so further sequencing analysis was required. Each band was gel isolated and cleaned (Nucleospin Gel & PCR Clean-up, Macherey-Nagel) for sequencing. In addition, the mixed RT–PCR product was cloned into the pCR 2.1 vector (TA cloning kit, Invitrogen) with blue/white selection (LB Amp, XGal, IPTG plates). Colony PCRs of white colonies (nine in total), using GoTaq (Promega) and universal M13 primers (M13F-20 GTAAAACGACGGCCAG, M13R CAGGAAACAGCTATGACC), were conducted using PCR cycling conditions: 95 °C 3 min, 31× (95 °C 30 s, 46 °C 30 s, 72 °C 35 s), and 72 °C 5 min. Clones with inserts were sent for sequencing with the M13 primers (Source BioScience). All sequences were checked for ambiguities, trimmed and aligned in BioEdit (RRID:SCR_007361), and translated with ExPASy (Swiss Bioinformatics Resource Portal). Of nine samples sequenced, three retained the ‘intron 1’ sequence, indicating that the double band from RT–PCR can be explained by alternative splicing. Because of the relative positions of a stop codon in the unprocessed intron sequence and the start of the 2.4 deletion just downstream (also creating a stop codon), the alternative splicing has a negligible effect on the encoded protein sequence compared with the *Ptcdpk2.4* deletion (two amino acids substituted, and an extra two amino acids deleted). In addition, we found a small variable region near the intron boundary (with a single nucleotide polymorphism, a 1 bp insert, and deletions of 8 bp or 21 bp observed). Translation of *Ptcdpk2.4* cDNA sequences indicated that all variants would produce severely truncated proteins (74–141 amino acids, compared with the full length of 538). All are predicted to be inactive.

### Generation of and Ca^2+^ signalling experiments with *Ptcdpk2* mutants expressing R-GECO1–mTurquoise

The WT RGMT strain was as generated and described by Harrison *et al*. (2025). *Ptcdpk2.1* and *Ptcdpk2.4* mutants were transformed with the RGECO1–mTurquoise_pPHAT1 via biolistic transformation with blasticidin (8 µg ml^–1^) selection (Helliwell *et al*., 2019). For the signalling experiments, cultures of transformed lines were grown for 4 d and used to inoculate the experiment plate. In a 24-well plate (Cat#. 83.3922, SARSTEDT) in a 2 ml volume, cells were inoculated at 30 000 cells ml^–1^, into either f/2 (-Si-vits) or low phosphate (f/2 -Si-vits, 1.8 µM Pi), with four replicates. The experiment cultures were then grown for 4 d. Wells were mixed and 160 µl of each replicate were added to a black 96-well plate (#655209 Greiner). The CLARIOstar Plus plate reader (BMG Labtech) was used to measure the R-GECO1 fluorescence at 547–15 nm/590–20 nm (excitation/emission) and the mTurquoise fluorescence at 434–15 nm/480–20 nm, over time in a well-wise manner, using the top optic. Each well was injected with either 40 µl of ASW+phosphate (resulting in 36 µM phosphate within the well) or ASW, as a control. The injection speed was 150 µl s^–1^. Wells were measured for ∼8 s before injection and then for ∼30 s after, with five flashes and an interval time of 0.69 s. This was performed on two separate plates for each replicate, so each strain was tested with four biological replicates and two technical replicates. Raw data were normalized by first dividing the R-GECO1 signal by the mTurquoise signal for all time points (R). Then the average R-GECO1/mTurquoise (R) value of all the measurements preceding the injection was used to calculate the R_0_ value. This was then used to divide all the other values (R/R_0_). The maximum R/R_0_ value was used to determine whether the cells responded to the injected stimulus; the maximum R/R_0_ value was averaged between technical replicates.

### Quantification of alkaline phosphatase activity

Alkaline phosphatase activity was quantified using the substrate *p*-nitrophenyl phosphate (pNPP) (Sigma-Aldrich, N7653) assay, which is based on the cleavage of pNPP by alkaline phosphatase resulting in a yellow substance. The protocol was modified from Shemi *et al*. (2016). Briefly, 1.5 ml of cells were centrifuged at 17 000 *g* for 5 min and resuspended in 176.9 μl of alkaline phosphatase buffer (0.01 M Tris pH 8, 0.05 M MgCl_2_, and 0.01 M CaCl_2_) with 23.1 µl of pNPP liquid substrate. Absorbance at 405 nm was measured at 2 min intervals during 30 min using a CLARIOstar Plus plate reader (BMG Labtech). Enzymatic activity was calculated using the change in absorbance over time, where the concentration&Δabsorbance at 405 nm/(path length×extinction coefficient). The extinction coefficient used was 18 000 M^−1^ cm^−1^, and path length was 0.57 cm.

### Quantifying phosphate concentrations in the media and calculating phosphate uptake rates

For phosphate detection in the media, a BIOMOL Green kit (Enzo Life Sciences, Germany) was used following the manufacturer’s protocol. Serial dilutions (1:1) of the phosphate standard in double-distilled H_2_O from the kit (800 μM) were made. The assay buffer was filtered seawater. Another standard using the phosphate from f/2 (36 μM) was used in a 1:1 serial dilution for comparison. Experimental samples were added to a 96-well plate and 100 μl of BIOMOL® GREEN reagent were added. The samples were left to incubate at room temperature for 30 min and read on a spectrophotometer plate reader at OD_620_ nm. Phosphate uptake rates were measured as previously described by Harrison *et al*. (2025). Briefly, a sample of medium was taken to confirm the initial level of phosphate, 500 µl of sample was removed and centrifuged (10 000 *g*, 5 min), and 200 µl of supernatant was retained for analysis. Then phosphate was resupplied to cultures of both WT and mutant lines, and 500 µl of sample was centrifuged (10 000 *g*, 5 min), and 200 µl of supernatant retained for analysis at increments between 0 min and 90 min. The rate of phosphate uptake was calculated by subtracting the phosphate detected in the media from 36 µM at the different time points, and calculating the change in phosphate in the cell fraction per minute. This was then normalized to the cell density to obtain pM per cell min^−1^.

## Results

### *P. tricornutum* CDPK repertoire, structure, and phylogeny

**Fig. 1.**
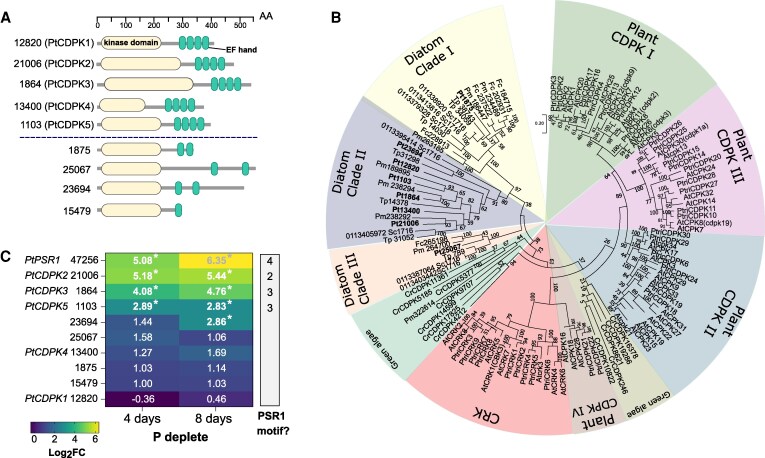
*In silico* analysis of *Phaeodactylum tricornutum* Ca^2+^-dependent protein kinases (CDPKs). (A) Domain analysis of CDPK-like hits in *P. tricornutum*. The kinase domain is in yellow and EF-hands are in green. The grey line represents the length of the protein. Protein identifiers for each protein are given. Domain analysis was performed using Interpro (Apweiler *et al*., 2000). As the gene encoding Pt12820 was previously annotated in the genome as PtCDPK1, Phatr2_21006 is referred to as PtCDPK2 in this study. Above the dotted line shows CDPKs with four EF-hands and below the line shows CDPK-like proteins with fewer than four EF-hands detected. (B) A maximum likelihood phylogenetic tree of CDPKs from *P. tricornutum* (*Pt*), *A. thaliana* (*At*), *Populus trichocarpa* (*Ptri*), *Chlamydomonas reinhardtii* (*Cr*), *Fragilariopsis cylindrus* (*Fc*), *Pseudo-nitzschia* multiseries (*Pm*), *Thalassiosira pseudonana* (*Tp*), and *Skeletonema costatum* (*Sc*). The tree was reconstructed using a WAG+Freqs (+F) correction model with 100 non-parametric bootstrap replicates, with bootstrap support indicated on the tree. (C) Heatmap showing log_2_fold changes in transcriptional expression of *P. tricornutum CDPK* hits during phosphate limitation for 4 d and 8 d compared with the 4-day-old old replete conditions, plotted from a previously published transcriptomic study (Cruz de Carvalho *et al*., 2016). Genes showing a significantly greater than 2-fold change (*P*-value <0.05) in P-depleted conditions are indicated with an asterisk. The numbers of PtPSR1 recognition motifs in the promoter region as reported by Sharma *et al*. (2020) are also indicated.

A targeted search for CDPKs in the *P. tricornutum* genome querying the functionally characterized *A. thaliana* AtCPK1 yielded nine CDPK hits containing both the necessary kinase and EF-hand domain(s) (Fig. 1A). Five of these hits had four predicted EF-hands typical of canonical CDPKs (Bredow and Monaghan, 2019), one of which (protein ID 12820) was annotated previously in the genome as PtCDPK1. The remaining four hits had kinase domains, but two or fewer predicted EF-hands. Surveying diatom genomes and transcriptomes more broadly, we also identified CDPKs in all pennate and centric species examined (Supplementary Fig. S5A; Supplementary Dataset S1). To determine the phylogenetic relationship of *P. tricornutum* CDPK-like sequences to a sample of homologues in other photosynthetic eukaryotes (including diatoms, plants, and green algae), an unrooted maximum-likelihood phylogenetic tree was reconstructed. This analysis revealed three distinct clades in diatoms (I–III), with at least one *P. tricornutum* CDPK hit in each clade. Clade II includes all five canonical CDPKs as well as Pt23694, none of which grouped with plant-specific CDPK or CDPK-related kinase (CRK) clades (Fig. 1B) (Bredow and Monaghan, 2022). Pt25067 and Pt1875, which each have two predicted EF-hands, belong to distinct diatom clades III (Pt25067) and I (Pt1875).

To investigate the potential role of the different *P. tricornutum* CDPKs during P limitation, we examined their expression in a previously published transcriptomic study (Cruz de Carvalho *et al*., 2016). Gene expression data in this analysis were available for all nine of the *P. tricornutum* CDPK hits. Three of these genes, *PtCDPK2*, *PtCDPK3*, and *PtCDPK5*, showed a significant (*P*<0.05) >2-fold increase in expression after 4 d and 8 d of P starvation relative to the 4-day-old replete control treatment (Fig. 1C) (Cruz de Carvalho *et al*., 2016). All have multiple PtPSR1 recognition motifs in their promoter regions (Sharma *et al*., 2020). Additionally, mining the DiatOmicBase portal (Villar *et al*., 2025), we found that *P. tricornutum* CDPKs show altered gene expression in response to a range of other environmental stressors (Supplementary Fig. S5B). This was also the case for *T. pseudonana* CDPKs, but notably one gene (protein ID 31052), which appears closely related to PtCDPK2, is significantly up-regulated in low P (Supplementary Fig. S5C).

### PtCDPK2 exhibits plasma membrane localization and is co-expressed with PtPSR1 under P limitation and resupply conditions

**Fig. 2.**
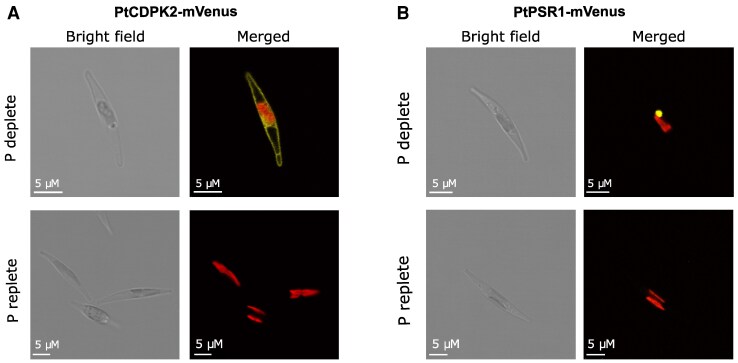
PtCDPK2 is plasma membrane localized and shows elevated expression in low-P conditions like PtPSR1. (A) Under P-depleted conditions (f/2 medium with 1.8 µM phosphate), PtCDPK2–mVenus is localized to the plasma membrane. No fluorescence was detected in P-replete conditions (36 µM phosphate). Both images were taken on day 4. (B) Expression and localization of PtPSR1–mVenus in P-depleted versus replete conditions (day 4).

As one of the most strongly up-regulated *PtCDPK* genes under P depletion, we further characterized PtCDPK2 by determining its subcellular localization, and examining its regulation by P availability, including in relation to PtPSR1. We compared transgenic lines expressing PtCDPK2–mVenus and PtPSR1–mVenus grown in P-replete (36 µM initial phosphate, as in f/2 medium) and previously defined P-limiting (1.8 µM initial phosphate) conditions (Helliwell *et al*., 2021a). PtCDPK2–mVenus was strongly expressed under P limitation, but no fluorescence was detectable in P-replete conditions (Fig. 2A). PtCDPK2–mVenus localized primarily to the cell periphery, suggesting an association with the plasma membrane. This was confirmed by co-staining with the membrane dye FM4-64 (Supplementary Fig. S6). PtCDPK2 does not have a transmembrane domain or the canonical plant MGXXXS/T motif for myristylation (Boudsocq and Sheen, 2013; Yip Delormel and Boudsocq, 2019), but it does have an MGXXS motif at position 38–42. In concurrence with transcriptional expression (Sharma *et al*., 2020), PtPSR1–mVenus was strongly up-regulated under P limitation and localized to the nucleus (Fig. 2B), as previously reported (Harrison *et al*., 2025).

**Fig. 3.**
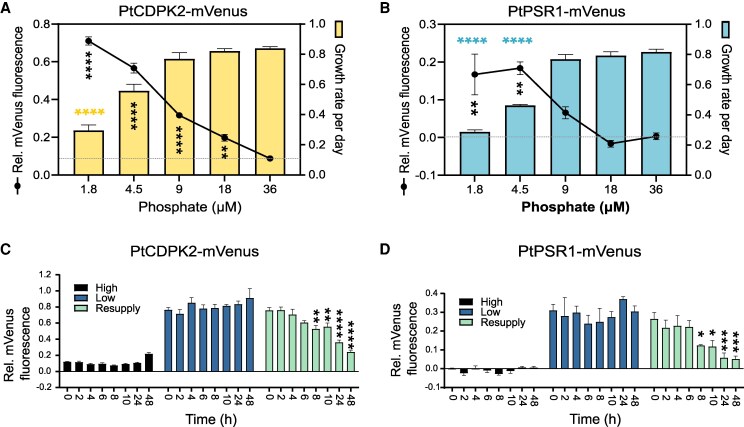
PtCDPK2 is co-expressed with PtPSR1 according to P availability. (A and B) Growth rate (d^−1^) between day 4 and 5 and relative mVenus fluorescence (mVenus/chlorophyll fluorescence) on day 5 for the PtCDPK2–mVenus (A) and PtPSR1–mVenus (B) strains. Data are means ±SE. Statistically significant results of a one-way ANOVA comparing the growth rate (d^−1^) of each phosphate treatment with the 36 µM control via a Dunnett’s multiple comparisons test, are indicated with coloured horizontal asterisks (****P<0.0001; ***P<0.001; **P<0.01; *P<0.05). The same test was also performed on the relative mVenus fluorescence data, and the results are shown with black vertical asterisks. (C and D) Time-course of relative mVenus fluorescence (mVenus/chlorophyll fluorescence) of PtCDPK2–mVenus (C) and PtPSR1–mVenus (D) strains grown under P-replete (36 µM phosphate) and P-depleted (1.8 µM phosphate) conditions, and following resupply of 36 µM phosphate to 4-day-old depleted cells. Results of a two-way ANOVA are indicated, comparing relative mVenus fluorescence at each time-point with that at time 0 (i.e. prior to P resupply to the resupply cultures) for each strain within the different P treatments (****P<0.0001; ***P<0.001; **P<0.01; *P<0.05).

We next measured the expression of PtCDPK2–mVenus and PtPSR1–mVenus over time when inoculated into media with different starting concentrations of phosphate. Increases in relative fluorescence were clearly visible by day 4 in treatments with initial phosphate concentrations of 1.8 µM and 4.5 µM (Supplementary Fig. S7). For higher concentrations, including 9 µM and 18 µM, there was no increase in relative mVenus fluorescence at day 4, but increases were seen at later time points. Even with 36 µM phosphate, some mVenus expression was detectable by day 7 for PtCDPK2–mVenus. In contrast, relative fluorescence of control WT cells remained around the baseline (of zero) for all phosphate concentrations throughout the experiment (Supplementary Fig. S7). Examining growth rate (d^−1^) versus mVenus fluorescence for the two strains across the range of phosphate concentrations tested demonstrated that PtCDPK2–mVenus and PtPSR1–mVenus were most strongly expressed when growth was P limited (Fig. 3A, B). However, for PtCDPK2–mVenus, significant increases in relative fluorescence (*P*-value <0.01, one-way ANOVA) were also evident even when there were no significant declines in growth rate compared with the 36 µM phosphate condition (i.e. 9 µM and 18 µM treatments; Fig. 3A).

Having demonstrated strong increases in PtCDPK2–mVenus and PtPSR1–mVenus under P deficiency, we monitored fluorescence following phosphate resupply. Significant reductions by 30% and 54% for PtCDPK2–mVenus and PtPSR1–mVenus, respectively, were observed within 8 h of phosphate resupply to 4-day-old 1.8 µM-grown cells (*P*<0.05, two-way ANOVA) (Fig. 3C, D). Values were comparable with those of replete cells within 48 h. To investigate the Ca^2+^ dependency of this response, we applied a pharmacological inhibitor of the P–Ca^2+^ signalling response, RuR (5 µM) (Helliwell *et al*., 2021a), to P-depleted cells prior to phosphate resupply on day 4 (Supplementary Fig. S8A, B). Replete and depleted cells were also examined in the presence and absence of RuR as controls. Decreases in PtCDPK2–mVenus fluorescence were observed after 6 h and 24 h following phosphate resupply in –RuR and +RuR pre-treated cells in a similar manner (Supplementary Fig. S8C, D). This indicates that the regulation of PtCDPK2 following phosphate resupply to P-limited cells is not dependent on P–Ca^2+^ signalling.

### A threshold intracellular P level defines activation of P–Ca^2+^ signalling

**Fig. 4.**
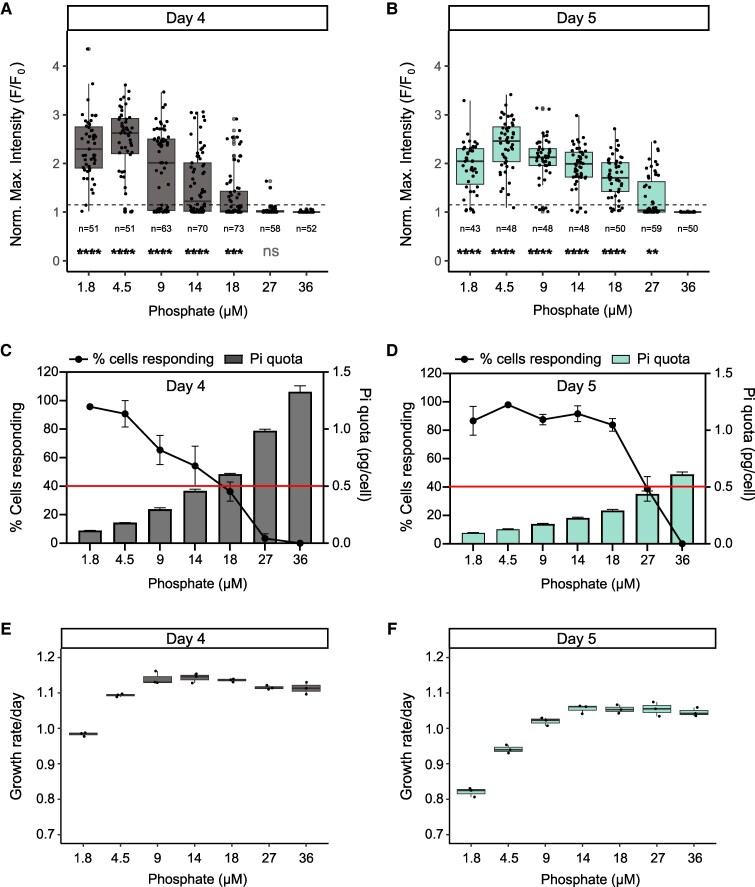
A threshold intracellular P level defines activation of P–Ca^2+^ signalling. (A and B) Mean normalized maximal fluorescence (*F*/*F*_0_) of PtR1 cells grown in different starting concentrations of phosphate and resupplied with 36 µM phosphate on day 4 (A) and day 5 (B). The horizontal dashed grey line indicates the 1.15 *F*/*F*_0_ threshold used to define whether a cell was deemed to have responded. Data are compiled from three independent replicate experiments per treatment, and the total number of cells examined per treatment is indicated on the figure. Results of a non-parametric Kruskal–Wallis test comparing each treatment with the P-replete 36 µM control are also given: ***P<0.0001; ***P<0.001; **P<0.01; *P<0.05; ‘ns’ not significant. (C and D) Phosphate quota per cell (pg per cell) after 4 d (C) and 5 d (D), respectively (calculated by dividing the initial phosphate concentration by the number of cells). The percentage of cells displaying a phosphate-induced Ca^2+^ signalling response is also shown (black line). Cells were deemed to have responded if the [Ca^2+^]_cyt_ fluorescence signal was above the *F*/*F*_0_ threshold of 1.15. The red line indicates where 40% of cells responded. Data are presented as the mean ±SE (*n*=3). (E and F) Growth rate (d^−1^) between day 0 and 4 (E) and 0 and 5 (F) of the PtR1 strain in the different phosphate treatments. Data are presented as the mean ±SE (*n*=3).

Phosphate-induced Ca^2+^ signals are also apparent only in P-depleted and not in P-replete cells (Helliwell *et al*., 2021a). As PtCDPK2 may play a role in sensing Ca^2+^ elevations generated by phosphate resupply, we examined whether P–Ca^2+^ signalling is activated by P starvation in a similar manner to PtCDPK2 expression (i.e. is PtCDPK2 only expressed under conditions where P–Ca^2+^ signalling is observed?). We grew PtR1 cells expressing the Ca^2+^ indicator R-GECO1 in f/2 medium with different initial phosphate concentrations. The capacity of cells for phosphate-induced Ca^2+^ signalling was then examined via Ca^2+^ imaging, resupplying 36 µM phosphate on days 4 and 5. After 4 d, no cells in the 36 µM phosphate treatment responded to phosphate resupply, with only a small percentage responding at the slightly lower concentration of 27 µM (Fig. 4A). A significant increase in the normalized mean maximal fluorescence intensity (*F*/*F*_0_) in response to phosphate resupply compared with the 36 µM replete control was detectable for all treatments with ≤18 µM of initial phosphate (Kruskal–Wallis test, *P*-values <0.001). By day 5, there was a significant increase (*P*-value <0.01) in normalized mean maximal *F*/*F*_0_ even in 27 µM phosphate-grown cells (Fig. 4B). Notably, exogenous phosphate was undetectable in all treatments by day 4, including the 36 µM control. This indicates that exhaustion of phosphate from the external environment does not trigger activation of the capacity of cells for P–Ca^2+^ signalling. We calculated cellular phosphate quotas by dividing the initial phosphate concentration by cell number, since all the phosphate had been taken up by the cells. Phosphate quotas per cell declined with decreasing levels of phosphate in the initial medium, as would be expected (Fig. 4C, D). Notably, levels needed to deplete to ∼0.5 pg of phosphate per cell for cells to exhibit P–Ca^2+^ signalling on both day 4 and 5. Together, this demonstrates that a threshold intracellular P level defines activation of the capacity for P–Ca^2+^ signalling and probably other P signalling responses. Moreover, activation of the ability for P–Ca^2+^ signalling occurred before limitation of growth rate by P (Fig. 4E, F), in a manner similar to increases in PtCDPK2–mVenus expression.

### *Ptcdpk2* mutants show increased physiological stress under phosphate limitation

To investigate the functional role of *PtCDPK2* in responding to P depletion and subsequent resupply, mutant lines were generated using CRISPR/Cas9 gene editing. Two independent mutants (*Ptcdpk2.1* and *Ptcdpk2.4*) were identified in which both the kinase and EF-hand domains of the protein were disrupted (Supplementary Fig. S9). *Ptcdpk2.1* possesses an insertion of 109 bp, whereas *Ptcdpk2.4* has a deletion of 546 bp (Supplementary Fig. S9A, B). Sanger sequencing of cDNA generated using RT–PCR indicated that the insertion in the coding sequence of *Ptcdpk2.1* causes a frameshift and in-frame stop codon, severely truncating PtCDPK2 (Supplementary Fig. S9C, D). For *Ptcdpk2.4*, more than one transcript was detected, indicating alternative splicing, but all variants analysed were predicted to cause severe PtCDPK2 truncation (Supplementary Fig. S9C, D; see the Materials and methods).

**Fig. 5.**
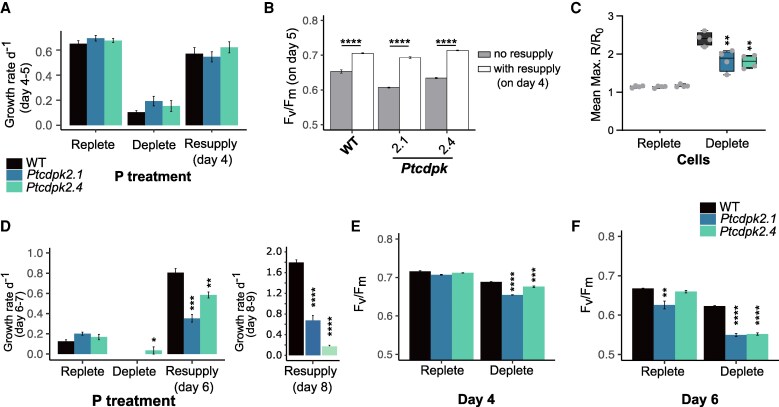
*Ptcdpk2* mutants show increased physiological stress under P limitation. (A) Growth rate (d^−1^) between day 4 and 5 for samples grown under replete (36 µM) and depleted (1.8 µM) conditions, and following resupply of 36 µM phosphate to 4-day-old depleted cells. Data are presented as the mean ±SE (*n*=3). A two-way ANOVA was performed on growth rates of mutants compared with the WT within each different treatment, but the data were not significant. (B) *F*_v_/*F*_m_ values of cultures on day 5 with or without resupply on day 4. Data are presented as the mean ±SD (*n*=3). Results of a two-way ANOVA comparing the resupply versus no resupply treatment for each line: *P <0.05, **P <0.01, ***P <0.001, ****P <0.0001. (C) Mean maximal fluorescence intensity (R/R_0_) of bulk populations (see the Materials and methods) of WT, *Ptcdpk2.1*, and *Ptcdpk2.4* cells transformed with the Ca^2+^ indicator R-GECO1–mTurquoise (RGMT) and grown in phosphate-replete (36 µM phosphate) or depleted (1.8 µM phosphate) conditions for 4 d, and then resupplied with 36 µM phosphate. Results of a one-way ANOVA (*n*=4 biological replicates) are shown: **P <0.01, comparing mutants with the WT within each treatment. Example traces for these data are shown in Supplementary Fig. S5. (D) As in (A), but growth rate (d^−1^) between day 6 and 7, with phosphate resupplied on day 6 instead of day 4. The inset on the right shows data from a separate experiment, resupplying phosphate to low-P-grown cells on day 8. Data are presented as the mean ±SE (*n*=3). Results of a one-way ANOVA and post-hoc Tukey test are shown, comparing growth of mutants versus the WT within each different treatment: *P <0.05, **P <0.01, ***P <0.001, ****P <0.0001. (E) *F*_v_/*F*_m_ of the WT compared with mutants in cultures inoculated into replete (36 µM phosphate) or depleted (1.8 µM phosphate) conditions on day 4. Data are presented as the mean ±SE (*n*=3). Results of a two-way ANOVA comparing each *Ptcdpk2* mutant with the WT within each treatment: *P <0.05, **P <0.01, ***P <0.001, ****P <0.0001. (F) As in (E), but on day 6.

To examine physiological impacts of functional knockout of *PtCDPK2*, *Ptcdpk2.1* and *Ptcdpk2.4* were grown under P-replete and depleted conditions (36 µM and 1.8 µM of initial phosphate, respectively) and following phosphate resupply on day 4. Growth dynamics of *Ptcdpk2* mutants and the WT were comparable between P regimes, with no statistically significant differences detected in the growth rate of mutants in low or high phosphate regimes between day 4 and 5 (Fig. 5A). Following phosphate resupply, the growth rate again did not differ between the WT and mutants, suggesting similar growth recovery capacity across all strains. The mutants also showed a significant recovery of photosynthetic efficiency (*F*_v_/*F*_m_) values within 24 h of phosphate resupply, like the WT (*P*-value <0.0001; two-way ANOVA, Fig. 5B). To investigate whether *Ptcdpk2* mutants are capable of the P–Ca^2+^ signalling response itself, mutant and WT lines were transformed with the Ca^2+^ biosensor RGMT (see the Materials and methods). Ca^2+^ signalling experiments demonstrated that both mutants were capable of the phosphate resupply-induced Ca^2+^ signalling response, although the maximal intensity was slightly reduced compared with the WT (Supplementary Fig. S10; Fig. 5C).

Whilst we observed no noticeable impairments in the ability of low-P-grown cells to recover growth or *F*_v_/*F*_m_ following phosphate resupply on day 4, during the course of our experiments, we noticed that both mutants showed significantly impaired growth rate recovery if phosphate was resupplied at a later time point (i.e. on day 6; Fig. 5D). This effect was even more significant when resupplying phosphate on day 8. To better understand this, we compared *F*_v_/*F*_m_ values of mutants versus the WT in P-replete and depleted conditions, on day 4 and 6. We observed that *F*_v_/*F*_m_ values of depleted cultures were significantly lower in *Ptcdpk2.1* and *Ptcdpk2.4* compared with the WT, and this effect became most pronounced by day 6 (Fig. 5E, F). No statistically significant differences were observed between the WT and *Ptcdpk2* mutants grown in replete conditions on day 4 (Fig. 5E; two-way ANOVA). However, we observed a significant reduction in *F*_v_/*F*_m_ of *Ptcdpk2.1* compared with the WT on day 6 for 36 µM-grown cells (Fig. 5F). Data from the previous experiment indicate that 36 µM-grown cells at day 5 are near the threshold of intracellular P depletion for induction of P–Ca^2+^ signalling (Fig. 4D) and are probably experiencing P starvation by day 6. Together these experiments demonstrate that *Ptcdpk2* mutants are defective in their ability to cope with P limitation. When experiencing more severe P depletion (i.e. after ≥6 d in low P medium), cells are less able to recover growth following phosphate resupply, but this is because the *Ptcdpk2* mutants are experiencing enhanced physiological stress prior to resupply compared with the WT.

### Examining P scavenging and uptake capacity of *Ptcdpk2* mutants compared with the wild type

**Fig. 6.**
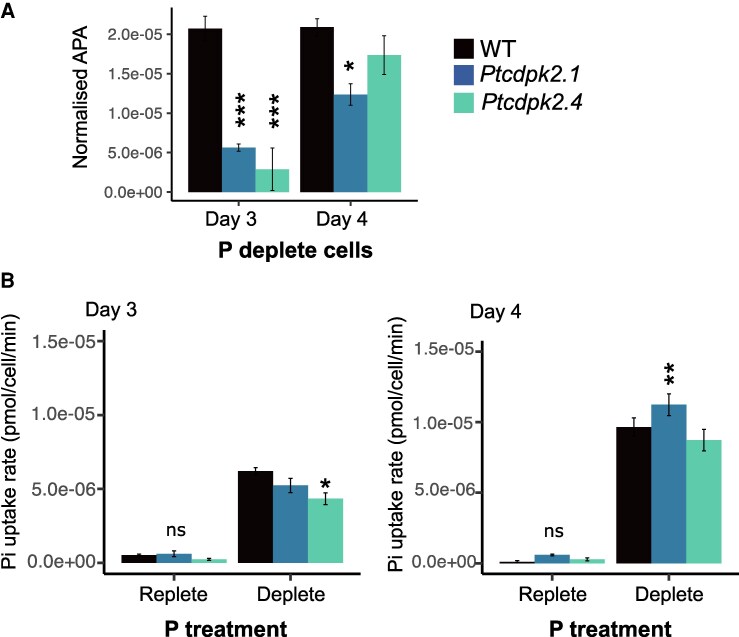
*Ptcdpk2* mutants show impaired alkaline phosphatase activity during P starvation. (A) Alkaline phosphatase activity (APA) (pNPP fmol min^–1^) normalized to chlorophyll fluorescence of cultures grown in f/2 medium with low phosphate (1.8 µM) after 3 d and 4 d. Data are presented as the mean ±SE (*n*=3). Data were tested for normality using the Shapiro test, and a one-way ANOVA was performed comparing mutants with the WT with Tukey post-hoc test: *P <0.05, ***P <0.001. (B) Phosphate uptake rates (pmol cell^–1^ min^–1^) of 3-day-old (left) and 4-day-old (right) cells grown in P-replete (36 µM phosphate) and P-depleted (1.8 µM phosphate) conditions. Results of a one-way ANOVA comparing mutants with the WT control are shown: *P <0.05, **P <0.01). Data are presented as the mean ±SE (*n*=3).

To further investigate what might be causing the reduced photosynthetic health and growth rate recovery of mutants following P resupply compared with the WT, we examined P scavenging (alkaline phosphatase activity) and uptake capacity in the different strains. *Ptcdpk2.1* and *Ptcdpk2.4* showed a 72.8% and 86.1% decrease in alkaline phosphatase activity, respectively, compared with the WT, after 3 d in low P media (*P*-value <0.001; one-way ANOVA, Fig. 6A). Whilst the effect was less pronounced by day 4, enzyme activity remained significantly lower for *Ptcdpk2.1.* As no significant differences in the growth rate of mutants versus the WT were observed in these conditions (Fig. 5A), the changes in enzyme activity are driven by an impaired ability of mutants to activate alkaline phosphatase under P depletion, rather than being due to differences in growth stage. This is likely to account for the increased physiological stress exhibited by the mutants compared with the WT in low P (Fig. 5). By comparison, differences in the phosphate uptake capacity of the mutants versus the WT were less pronounced. No differences were seen in the uptake capacity of strains grown in P-replete conditions. For cells grown in low P, a slight (albeit significant) difference was detected between the WT and *Ptcdpk2.4* on day 3, as well as *Ptcdpk2.1* on day 4, but consistent trends were not observed across both mutants (*P*-value <0.05; one-way ANOVA, Fig. 6B).

## Discussion

CDPKs have been studied in divergent eukaryotes including plants, green and red algae (Valmonte *et al*., 2014; Brawley *et al*., 2017), and heterotrophic protists (Billker *et al*., 2009; Sharma *et al*., 2021). Here, we report a comprehensive examination of CDPKs in the diatom *P. tricornutum*. We identified five canonical *CDPK* genes, encoding both the expected kinase domain as well as four EF-hands (Harper *et al*., 2004; Bredow and Monaghan, 2022) (Fig. 1). *Phaeodactylum tricornutum* CDPKs were phylogenetically distinct from plant and green algal CDPKs (Li *et al*., 2019). Three *P. tricornutum CDPK* genes show strong transcriptional up-regulation by P limitation (Cruz de Carvalho *et al*., 2016; Sharma *et al*., 2020; Villar *et al*., 2025), and contain PtPSR1 recognition motifs in their promoter regions (Sharma *et al*., 2020). In this study, we focused on characterizing *PtCDPK2*. We report that PtCDPK2 localizes to the cell periphery and is strongly induced by P limitation (Figs 2, 3), in a manner similar to the P–Ca^2+^ signalling response (Fig. 4). Expression is also rapidly down-regulated within 8 h following phosphate resupply, like PtPSR1 that probably regulates *PtCDPK2* (Sharma *et al*., 2020). We hypothesized that PtCDPK2 may be involved in sensing P-induced Ca^2+^ elevations and coordinating recovery responses of P-limited *P. tricornutum* cells. However, our collective findings support a primary role for PtCDPK2 in the regulation of P starvation physiology, with *Ptcdpk2* mutants showing significantly reduced *F*_v_/*F*_m_ (Fig. 5) and alkaline phosphatase activity under P depletion (Fig. 6). The poor health of *Ptcdpk2* mutants after prolonged P limitation affects their ability to recover growth following P resupply. Hence, we propose that PtCDPK2 confers a second tier, or outpost of PtPSR1, in regulating *P. tricornutum* P starvation responses.

CDPK diversity increases with morphological complexity in plants and green algae (Edel *et al*., 2017). *Chlamydomonas reinhardtii* encodes 15 CDPKs, whereas *A. thaliana* has 34 (Yip Delormel and Boudsocq, 2019). We identified a broad range in the number of CDPKs in diatoms, with many species possessing more than multicellular plants (e.g. compared with the pteridophyte *Selaginella moellendorffii* and gymnosperm *Pinus taeda* that have 10 and 13, respectively) (Supplementary Fig. S5; Supplementary Dataset S1). Many diatoms also have more CDPKs than multicellular brown seaweed relatives (e.g. 16 in *Saccharina japonica*) (Sun *et al*., 2025). Hence, factors beyond the evolution of multicellularity are clearly important in shaping CDPK gene family expansion. As diatoms arose via secondary endosymbiosis, their complex evolutionary history could be an additional factor, as well as their adaptation to diverse habitats and lifestyles. Functional redundancy has hindered study of plant CDPKs (Liu *et al*., 2017; Yip Delormel and Boudsocq, 2019). It is possible that the other CDPKs up-regulated in *P. tricornutum* under P deficiency could perform redundant roles in *Ptcdpk2* mutants. However, complete compensation did not occur since we observed convincing reductions in *F*_v_/*F*_m_ and alkaline phosphatase activity in the mutants compared with the WT under P limitation. Interestingly, these effects did not lead to detectable reductions in the growth rates of mutants versus the WT in low-P conditions. This is probably because growth has virtually ended in these conditions in any case. Similarly, no significant differences in growth rate were detectable for alkaline phosphatase (PhoD) mutants compared with the WT in low-P conditions (Li *et al*., 2022). However, because *Ptcdpk2* mutants are less able to regulate adaptations to P starvation, including a decreased ability to scavenge organic P forms, cells become more stressed when P is scare. This probably caused the greater declines in *F*_v_/*F*_m_ observed for mutants versus the WT, and their reduced ability to recover growth following phosphate resupply after more prolonged periods of P starvation. Moreover, although *Ptcdpk2* mutants were capable of P–Ca^2+^ signalling, we hypothesize that the slight reduction in maximal signal intensity observed in mutants compared with the WT, was also due to their poorer cell health under P starvation since P depletion status strongly influences P–Ca^2+^ signal amplitude (Fig. 4). However, this warrants further investigation.

An additional challenge for characterizing other *P. tricornutum* CDPKs going forward is the potential for gene knockout lethality. The CDPK repertoire of *P. tricornutum* is more comparable in size with apicomplexan protists (Keeling and Burki, 2019). *Plasmodium falciparum* has seven and *Toxoplasma gondii* ∼12 CDPKs (Patil *et al*., 1995; Billker *et al*., 2009). Apicomplexan CDPKs can be essential for parasite viability, necessitating the use of conditional knockout and/or chemical inhibition for functional gene characterization (Kato *et al*., 2008; Lourido *et al*., 2010). Integrative techniques combining protein localization, reverse genetics, and phosphoproteomics (Liu *et al*., 2020) will help shed further light on the roles of diatom CDPKs. However molecular tool development enabling conditional knockout is also needed to allow generation of double and triple mutants and help overcome the potential for gene knockout lethality/redundancy in diatoms.

The localization of PtCDPK2 to the cell periphery strongly suggests a primary role regulating membrane processes. *Phaeodactylum tricornutum* encodes eight alkaline phosphatases and 10 phosphate transporters localized to different regions of the cell including the plasma membrane, like PtCDPK2 (Dell’Aquila *et al*., 2020). The alkaline phosphatases PtPhos8 and PtPhos3 are found in (but not limited to) the plasma membrane (Dell’Aquila *et al*., 2020), whereas PtPhos5, PtPhos6, and PtPhos7 show endomembrane localization. Additionally, PtPhos1 and PtPhos2 are secreted under P deficiency (Lin *et al*., 2013, 2017; Buhmann *et al*., 2016; Dell’Aquila *et al*., 2020). This is hypothesized to be modulated by protein phosphorylation since different mass variants of PtPHOS1 have been detected (Dell’Aquila *et al*., 2020), but the specific phosphosites and kinase(s) responsible are yet to be elucidated. Our data point to PtCDPK2 as a strong candidate. Nevertheless, significant redundancy in diatom alkaline phosphatases also introduces potential for phenotype compensation that could explain why the alkaline phosphatase phenotype became less pronounced with time in P-depleted conditions. Functional complementation of PtPhos1 by PtPhos6, and vice versa, has been reported previously in *P. tricornutum* (Zhang *et al*., 2022).

The ability to cope with fluctuating P supply and regulate P starvation physiology accordingly demands molecular mechanisms to sense P availability. In *Saccharomyces cerevisiae*, internal P levels are sensed via SPX (SYG1/Pho81/XPR1) proteins that bind inositol phosphates, triggering synthesis of poly-P (Lonetti *et al*., 2011; Wild *et al*., 2016; Gerasimaite *et al*., 2017). Diatoms have SPX proteins (Zhang *et al*., 2021), as well as vacuolar transport chaperone homologues (PtVTC 1–PtVTC4) (Schreiber *et al*., 2017). However, diatoms employ PSR1, instead of Pho80/Pho85/Pho4, for P starvation regulation. Employing a reporter gene approach, we have been able to study with high temporal resolution the regulation of PtPSR1 and PtCDPK2 by P availability, in relation to P–Ca^2+^ signalling, and internal and external P levels. Our collective evidence indicates that growth need not be P limited, and a threshold cellular P level, rather than exhaustion of external P, defines activation of P signalling. These dynamics must next be examined in the context of cellular P reserves, such as poly-P. Lapointe *et al*. (2024) recently visualized poly-P granules in the vacuoles of *Achnanthidium minutissimum*, a freshwater diatom. *A. minutissimum* displays a remarkable capacity to take up phosphate, and rapidly accumulate poly-P. This so-called overplus response is common to cyanobacteria (Li and Dittrich, 2019) and green algae (Plouviez *et al*., 2021; Zúñiga-Burgos *et al*., 2024). Poly-P levels persisted for 2 d following inoculation of *A. minutissimum* into –P medium. Moreover, exponential growth was maintained despite P depletion for ∼10 d (Lapointe *et al*., 2024). This is testament to the attributes of diatoms for coping with P deprivation.

Central to the regulation of P starvation responses in divergent photosynthetic eukaryotes is PSR1 (Wykoff *et al*., 1999; Rubio *et al*., 2001; Sharma *et al*., 2020). Overexpression of CrPSR1 in *C. reinhardtii* induces luxury phosphate uptake (Slocombe *et al*., 2023). Moreover, in *P. tricornutum*, *Ptpsr1* mutants exhibit impaired P–Ca^2+^ signalling (Harrison *et al*., 2025). This evidence combined with the co-expression of PtCDPK2 with PtPSR1, and the presence of PtPSR1-binding motifs in upstream regions of *PtCDPK* genes (Sharma *et al*., 2020), suggests that PtPSR1 may regulate Ca^2+^ sensor machinery, which warrants further investigation. As evidence so far suggests that diatom-like P–Ca^2+^ signalling is not displayed by other photosynthetic eukaryotes (Pivato *et al*., 2025), hijacking of PSR1 for regulation of the Ca^2+^ signalling apparatus may have occurred in diatoms. This is not dissimilar to land plants, whereby plant mycorrhizal symbiosis genes have become wired to the PSR1 protein PHR2 in rice (Das and Gutjahr, 2022; Das *et al*., 2022). These examples highlight P nutrition as a powerful driver shaping the divergent biology and diverse strategies of photosynthetic eukaryotes to survive in environments with variable P availability. As a central hub regulating such processes, PSR1 may well provide an exciting gateway for discovery of novel genes underlying such biology. Our work illuminating a role for CDPKs in regulating diatom P starvation responses now also warrants further attention to identify downstream players and interacting partners.

## Supplementary Material

erag114_Supplementary_Data

## Data Availability

Open access transcriptome and genome datasets mined during this study are available via the DiatOmicBase portal. Cloning information including primers, guide RNAs, other relevant sequence details, and plasmid accession numbers are given in full in the Materials and methods and Supplementary data of this study. All other materials presented are available upon request.

## Supplementary data

The following supplementary data are available at *JXB* online.

Fig. S1. Gene model for *PtCDPK2* from *P. tricornutum* genome v3.

Fig. S2. Cloning information for the sgRNA cassette and recipient plasmid for *PtCDPK2* gene editing.

Fig. S3. DNA sequence of *Ptcdpk2.1*.

Fig. S4. DNA sequence of *Ptcdpk2.4*.

Fig. S5. CDPKs are abundant and widely distributed across pennate and centric diatoms.

Fig. S6. PtCDPK2–mVenus expression and localization under P-depleted conditions.

Fig. S7. PtCDPK2–mVenus and PtPSR1–mVenus are up-regulated under P starvation.

Fig. S8. Decreases in PtCDPK2–mVenus fluorescence following phosphate resupply to P-depleted cells are not dependent on P–Ca^2+^ signalling.

Fig. S9. Screening *Ptcdpk2* mutants.

Fig. S10. Examining the capacity of *Ptcdpk2* mutants for phosphate-induced Ca^2+^ signalling.

Dataset S1. Distribution and protein IDs of CDPK hits amongst representative diatoms from transcriptome and genome data.
