# Supplementary material for: A plasma membrane Ca2+-dependent protein kinase PtCDPK2 promotes phosphorus starvation resilience in Phaeodactylum tricornutum
Source: J Exp Bot. 2026 Feb 28;77(10):3245–60. doi: 10.1093/jxb/erag114 (PMC13187674; doi:10.1093/jxb/erag114)
Supplement: erag114_Supplementary_Data [file erag114_supplementary_data.zip › jexbot316736-file001.pdf]

Figure S1: Gene model for *PtCDPK2* (protein id. 21006), from *P. tricornutum* genome v3. To generate the PtCDPK2-mVenus construct this sequence was synthesised, excluding the TGA stop codon but including flanking restrictions site sequences for PstI (GGGCTGCAG) and StuI (AGGCCT). The DNA fragment was synthesised and cloned by Genscript (GenScript, Piscataway, NJ) into the pPha-T1-mVenus vector (a derivative of the pPHA-T1 (accession AF219942) adapted as described by (Helliwell et al., 2019) containing a codon optimised mVenus gene (AJN91098.1).

XX = Upstream untranslated region
XX = Exon
XX = Intron

>PtCDPK21006_ gene model
GTCTACAAAAGCATGTCTTGTAAGCTAATCCATCCTTTATAGATCAGGATTGGAATAACCTGCCAAAA
ACCTACTACCTATAGAGTTCAATTCTGGGTCAGCTTCTTTCAATGGTGACAGTAGTTCCGTCACATAA
AGCGGTGGCCACTTGTTTCTCCCTTCGTGGTGTCCGTGACTCTCAATTGTGAAAAACCCTCACTCTCT
GGTGAAAAGTGAAGAAAACCAGGGTAGAGTAGGAACTAGCAGGTTTCTTGCTGACAGCGAATCCAT
TCTTACGCTCATGTAGAGATGTGCAGTCTGAGGGGTTGGTCAAGAGAATGTTGCGGGGCCATGTTA
CACTCGTGGCGGTGTGTAGCGAATTTGTTTCCACTCATTGTAGAGAAAAACAACAAAGATAAAAAG
GAAAATCCACTATATGGGATACTACGATTCGCAGTCTTTTGAAGACCCGGAAATGAGAATCTCAGCG
TCCGTTGAGCGAATCTAACGTTAGGTTTCGAAATTTTGAAGTGATCGCGCCGCGTAGGAATCAGCAA
AAAACAGCCGAAGAAGATCCAAGCTGCCGACGACCGCTCGGCTCGCACAAGGAACGACCGAGTTCA
AGACAGAACCAGCAGCCAGAATGTTATGTAGACCTTGTTGTGACGAAGACAGCGACAAATCTGCTT
CTCGTAGCCTGTTCACACCACATATTCAAGCTATGGACGAACTTCCTATTACCGACCCTTCCATCCATG
CCAACCTCCTACTCGAATCGAAGGAGGATGTCTTCAAAAAATATTCCGTCGTCCAAGTGCTCGGTAA
TGGATCAATGGGAACCGTTTCCAAGGTCAAAATTAAGAAGCACAAGGTCGGGGGAAGCGCCTTTCA
GCCGAAATCCAAGGGAATTTTTGGCTTTTTGAAGAAACAGAACAACAAAAGGAAGGAAGGTGAGA
CCAGAGAACACAATAGTCAGGACTATATATACGCACTCAAGTCCATCATTCTGGATCGGGTCTCTTCT
GTCTTCCTGGACGAGCTCCGTAACGAAATTCTTATCCTTAGATCATTGGATCATCCCAATATTGTCAA
AGCGCACGAGGTTTACTACACGAGGAAGCAGATTTATCTCGGTGCGTGATGAGGAATGTGAAACGT
GATTGGTCAGAAGTCTGAACTTTTTCTCTTTGGAAAGGAGACTAACGCATTTCCGTCATCGTTTTCTT
ATATCCGTGTGCTGTTTCTGGAAGTATTGGAGTTGTGTGATGGCGGAGACCTTTATACCAGGTCGCC
TTACAGTGAAAGGGAATCGGCAAGGATTCTGCAACAAATATTGTCGGCAGTGCGGTACATGCATGG
TACGCTATACCGATACGCATATAGAATTCCCGCAGCAAACTTTGGGAAGCTAACAATTCTTGTTCTGC
TTTGTCTAGATCACGGAATTGTTCATCGGGATCTCAAGTTCGAGAATATCATGTTTGAGAACAATAG
CCCCAGTGCTCGGTAGGTTACTGATCAACTCTCGAGGCTACACGTAGATGAAGCGCTCTTACAGTCA
ATAATGTTCTATTCATTTTGCACAGAGTCAAAATTATAGATTTTGGATTGTCTAAAAAGTTCCTTGGC
AAACCGTCGTACATGACCGAACGCGTTGGTACCGTCTATACGATGGCCCCGCAAGTCCTGCAAGGA
GTCTACTCATCGCAAGCTGATCTTTGGTCCGCTGGAGTGATAGCCTACATGCTGTTATCGGCTTCAAA
GCCTTTTTATCACAAACGACGGCGCAAGATGATTGACCAAATCATGAGGGCCGACTTCGGATATAAT
GCACCGGTCTGGAAGCAAATATCAGAAAGTGCGCAAGATTTTGTAAGTCGATTACTAGTGGTGGAT
CCAAAGAAAAGACTGAATGCAGAAAAAGCATTGGACCATTCTTGGATTGTGAATCGCGAACGCTTG
CCAGATGAGACACCATCCGAGGATTTGTTGGCCGCTGTCGATGATTGCCTCGTGAATTATCGACAAA
CGTCGGAGCTGAAAAAGCTAGCTTTAAACATGATCGCCCATCGTTCTACCGCGGAAGAGATCATGCA
ACTTCGGAAAGTTTTTGACAGCTACGACACCTCGAATGATGGAATTATTACATTTGATGAATTCAAAG
CAGCTTTGCACAAAATGAAATATCCGGATGAGATTGTACAGGAAGTTTTTAGCAGTATTGATGTCAA
CCGAAATGGCCATATACAGTACACGGAATTCATTGCATCGACCGTCTTGGCACAGGGACATATCGCA

GAGGATCGGGTCGCAGTAGCTTTCGATCGCTTGGACTCTGATGACACCGGCTTTATTTCCAAGAAGA
ACTTGCAAAACGCATTGGGCAAGGAATACACTCCAGAACTCGTCGAAAATATAATGGAAGAAGTTG
ACAAAGATAGGGATGGCAAAATATCATATACCGAGTTTCTGCAATACTTTCGGAAGGAAACGAGCA
ATCTGGCCGACAGGGCCTCTCTTTTAGAGCAACAGTCGTCACATGTAAGCGAACACGGTCTGGTCGG
TTTGGACGCCAAGATTCCTGGAGGACCGTACGATCCTAACCGAACT**TGA**

Figure S2. Cloning information for the sgRNA cassette and recipient plasmid for *PtCDPK2* gene editing. Description and features of the DNA fragment synthesised, containing the CDPK 21006_dual sgRNA cassette cloned into pKSdiaCas9 (Nymark et al., 2016) using restriction site/enzyme BsaI. The plasmid map (Addgene, plasmid #74923) for pKSdiaCas9 is also given. Note the U6 promoter driving expression of sgRNA1_Pt21006, is present in pKSdiaCas9 (red) upstream of the first BsaI site. The resulting assembled plasmid was subsequently co-transformed into *P. tricornutum* with pPHAT1 (accession no.: AF219942) conferring resistance to zeocin to generate the *Ptcdpk2* mutants described in this paper.

XX = BsaI bind
XX = BsaI cut
XX = sgRNA1_Pt21006
XX = sgRNA2_Pt21006
XX = sgRNA Cas9bind
XX = Pt U6Promoter

>CDPK 21006_dual sgRNA cassette
GGTCTCATCGAGAAACAGAACAACAAAAGGAGTTTTAGAGCTAGAAATAGCAAGTTAAAATAAGGC
TAGTCCGTTATCAACTTGAAAAAGTGGCACCGAGTCGGTGCTTTTTTTCTAGACCCAGCTTTCTTGTA
CAAAGTTGGCATTACGCTTTACGAATTCCCATGGGGAGGTTGGCTCGGAAGTTGGTGTTGACGGTG
AGCTGGAAATTGGTTGTCGGTCACTGCTAGCGAGAAGAAAACGGAGGACAGAAGGAAGTGAAACT
CGGTTCGTTCTCGACAGCCTCACTGTCAATATGCTCATTTTCAATCCTTAGCGCTTTTAATGTCGAATT
GACGGTAAATTGAATAGGATCTATAATATCTACAAGGTACTTTGACACGCCAAGTATTCATTGTTAGT
CAACAATATTTTAGAGCTTTATAAGGTCAAAAAACACCTTCAAAGTCGAGGAAGTATTGGAGTTGTG
TGAGTTTAGAGACC

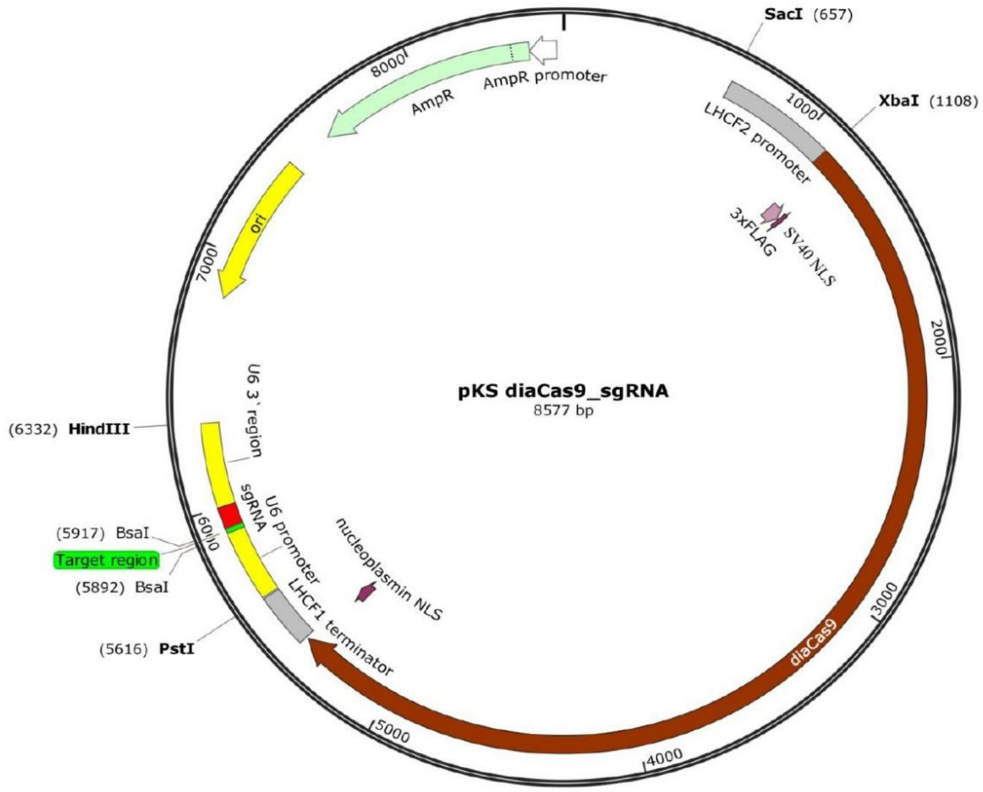

Figure S3: DNA sequence of *Ptcdpk2.1*

Insertion
XX = Exon
XX = Intron
XX= Primer binding sites for KEH_409F and KEH_410R

>Ptcdpk2.1
ATGGACGAACTTCCTATTACCGACCCTTCCATCCATGCCAACCTCCTACTCGAATCGAAGGAGGATGT
CTTCAAAAAATATTCCGT**CGTCCAAGTGCTCGGTAATG**GATCAATGGGAACCGTTTCCAAGGTCAAA
ATTAAGAAGCACAAGGTCGGGGGAAGCGCCTTTCAGCCGAAATCCAAGGGAATTTTTGGCTTTTTG
AAGAAACAGAACAACAAAAGGAAGGAAGGTGAGACCAGAGAACACAATAGTCAGGACTATATATA
CGCACTCAAGTCCATCATTCTGGATCGGGTCTCTTCTGTCTTCCTGGACGAGCTCCGTAACGAAATTC
TTATCCTTAGATCATTGGATCATCCCAATATTGTCAAAGCGCACGAGGTTTACTACACGAGGAAGCA
GATTTATCTCGGTGCGTGATGAGGAATGTGAAACGTGATTGGTCAGAAGTCTGAACTTTTTCTCTTT
GGAAAGGAGACTAACGCATTTCCGTCATCGTTTTCTTATATCCGTGTGCTGTTTCTGGAAGTATTGGA
GTTGTGTGAGAAAACTCACCTCTGCCTAGCCTACACGACGCTGAAGCTATGTGTCTCGAGAAAACTC
ATCCTGTGCCTTCCTCACTTTCTGCTAGTCCACTCCAACAATACGGCGTGATGGCGGAGACCTTTATA
CCAGGTCGCCTTACAGTGA**AAGGG**AATCGGCAAGGATTCTGCAACAAATATTGTCGGCAGTGCGGT
ACATGCATGGTACGCTATACCGATACGCATATAGAATTCCCGCAGCAAACTTTGGGAAGCTAACAAT
TCTTGTTCTGCTTTGTCTAGATCACGGAATTGTTCATCGGGATCTCAAGTTCGAGAATATCATGTTTG
AGAACAATAGCCCCAGTGCTCGGTAGGTTACTGATCAACTCTCGAGGCTACACGTAGATGAAGCGCT
CTTACAGTCAATAATGTTCTATTCATTTTGCACAGAGTCAAAATTATAGATTTTGGATTGTCTAAAAA
GTTCCTTGGCAAACCGTCGTACATGACCGAACGCGTTGGTACCGTCTATACGATGGCCCCGCAAGTC
CTGCAAGGAGTCTACTCATCGCAAGCTGATCTTTGGTCCGCTGGAGTGATAGCCTACATGCTGTTAT
CGGCTTCAAAGCCTTTTTATCACAAACGACGGCGCAAGATGATTGACCAAATCATGAGGGCCGACTT
CGGATATAATGCACCGGTCTGGAAGCAAATATCAGAAAGTGCGCAAGATTTTGTAAGTCGATTACTA
GTGGTGGATCCAAAGAAAAGACTGAATGCAGAAAAAGCATTGGACCATTCTTGGATTGTGAATCGC
GAACGCTTGCCAGATGAGACACCATCCGAGGATTTGTTGGCCGCTGTCGATGATTGCCTCGTGAATT
ATCGACAAACGTCGGAGCTGAAAAAGCTAGCTTTAAACATGATCGCCCATCGTTCTACCGCGGAAGA
GATCATGCAACTTCGGAAAGTTTTTGACAGCTACGACACCTCGAATGATGGAATTATTACATTTGAT
GAATTCAAAGCAGCTTTGCACAAAATGAAATATCCGGATGAGATTGTACAGGAAGTTTTTAGCAGTA
TTGATGTCAACCGAAATGGCCATATACAGTACACGGAATTCATTGCATCGACCGTCTTGGCACAGGG
ACATATCGCAGAGGATCGGGTCGCAGTAGCTTTCGATCGCTTGGACTCTGATGACACCGGCTTTATT
TCCAAGAAGAACTTGCAAAACGCATTGGGCAAGGAATACACTCCAGAACTCGTCGAAAATATAATG
GAAGAAGTTGACAAAGATAGGGATGGCAAAATATCATATACCGAGTTTCTGCAATACTTTCGGAAG
GAAACGAGCAATCTGGCCGACAGGGCCTCTCTTTTAGAGCAACAGTCGTCACATGTAAGCGAACAC
GGTCTGGTCGGTTTGGACGCCAAGATTCCTGGAGGACCGTACGATCCTAACCGAACT**TGA**

Figure S4: DNA sequence of *Ptcdpk2.4*

Region deleted
XX = Exon
XX = Intron
XX= Primer binding sites for KEH_409F and KEH_410R

>Ptcdpk2.4
ATGGACGAACTTCCTATTACCGACCCTTCCATCCATGCCAACCTCCTACTCGAATCGAAGGAGGATGT
CTTCAAAAAATATTCCGT**CGTCCAAGTGCTCGGTAATG**GATCAATGGGAACCGTTTCCAAGGTCAAA
ATTAAGAAGCACAAGGTCGGGGGAAGCGCCTTTCAGCCGAAATCCAAGGGAATTTTTGGCTTTTTG
AAGAAACAGAACAACAAAAGGAAGGAAGGTGAGACCAGAGAACACAATAGTCAGGACTATATATA
CGCACTCAAGTCCATCATTCTGGATCGGGTCTCTTCTGTCTTCCTGGACGAGCTCCGTAACGAAATTC
TTATCCTTAGATCATTGGATCATCCCAATATTGTCAAAGCGCACGAGGTTTACTACACGAGGAAGCA
GATTTATCTCGGTGCGTGATGAGGAATGTGAAACGTGATTGGTCAGAAGTCTGAACTTTTTCTCTTT
GGAAAGGAGACTAACGCATTTCCGTCATCGTTTTCTTATATCCGTGTGCTGTTTCTGGAAGTATTGGA
GTTGTGTGATGGCGGAGACCTTTATACCAGGTCGCCTTACAGTGAAAGGGAATCGGCAAGGATTCT
GCAACAAATATTGTCGGCAGTGCGGTACATGCATGGTACGCTATACCGATACGCATATAGAATTCCC
GCAGCAAACTTTGGGAAGCTAACAATTCTTGTTCTGCTTTGTCTAGATCACGGAATTGTTCATCGGGA
TCTCAAGTTCGAGAATATCATGTTTGAGAACAATAGCCCCAGTGCTCGGTAGGTTACTGATCAACTCT
CGAGGCTACACGTAGATGAAGCGCTCTTACAGTCAATAATGTTCTATTCATTTTGCACAGAGTCAAA
ATTATAGATTTTGGATTGTCTAAAAAGTTCCTTGGCAAACCGTCGTACATGACCGAACGCGTTGGTA
CCGTCTATACGATGGCCCCGCAAGTCCTGCAAGGAGTCTACTCATCGCAAGCTGATCTTTGGTCCGC
TGGAGTGATAGCCTACATGCTGTTATCGGCTTCAAAGCCTTTTTATCACAAACGACGGCGCAAGATG
ATTGACCAAATCATGAGGGCCGACTTCGGATATAATGCACCGGTCTGGAAGCAAATATCAGAAAGT
GCGCAAGATTTTGTAAGTCGATTACTAGTGGTGGATCCAAAGAAAAGACTGAATGCAGAAAAAGCA
TTGGACCATTCTTGGATTGTGAATCGC**GAACGCTTGCCAGATGAGAC**ACCATCCGAGGATTTGTTGG
CCGCTGTCGATGATTGCCTCGTGAATTATCGACAAACGTCGGAGCTGAAAAAGCTAGCTTTAAACAT
GATCGCCCATCGTTCTACCGCGGAAGAGATCATGCAACTTCGGAAAGTTTTTGACAGCTACGACACC
TCGAATGATGGAATTATTACATTTGATGAATTCAAAGCAGCTTTGCACAAAATGAAATATCCGGATG
AGATTGTACAGGAAGTTTTTAGCAGTATTGATGTCAACCGAAATGGCCATATACAGTACACGGAATT
CATTGCATCGACCGTCTTGGCACAGGGACATATCGCAGAGGATCGGGTCGCAGTAGCTTTCGATCG
CTTGGACTCTGATGACACCGGCTTTATTTCCAAGAAGAACTTGCAAAACGCATTGGGCAAGGAATAC
ACTCCAGAACTCGTCGAAAATATAATGGAAGAAGTTGACAAAGATAGGGATGGCAAAATATCATAT
ACCGAGTTTCTGCAATACTTTCGGAAGGAAACGAGCAATCTGGCCGACAGGGCCTCTCTTTTAGAGC
AACAGTCGTCACATGTAAGCGAACACGGTCTGGTCGGTTTGGACGCCAAGATTCCTGGAGGACCGT
ACGATCCTAACCGAACT**TGA**

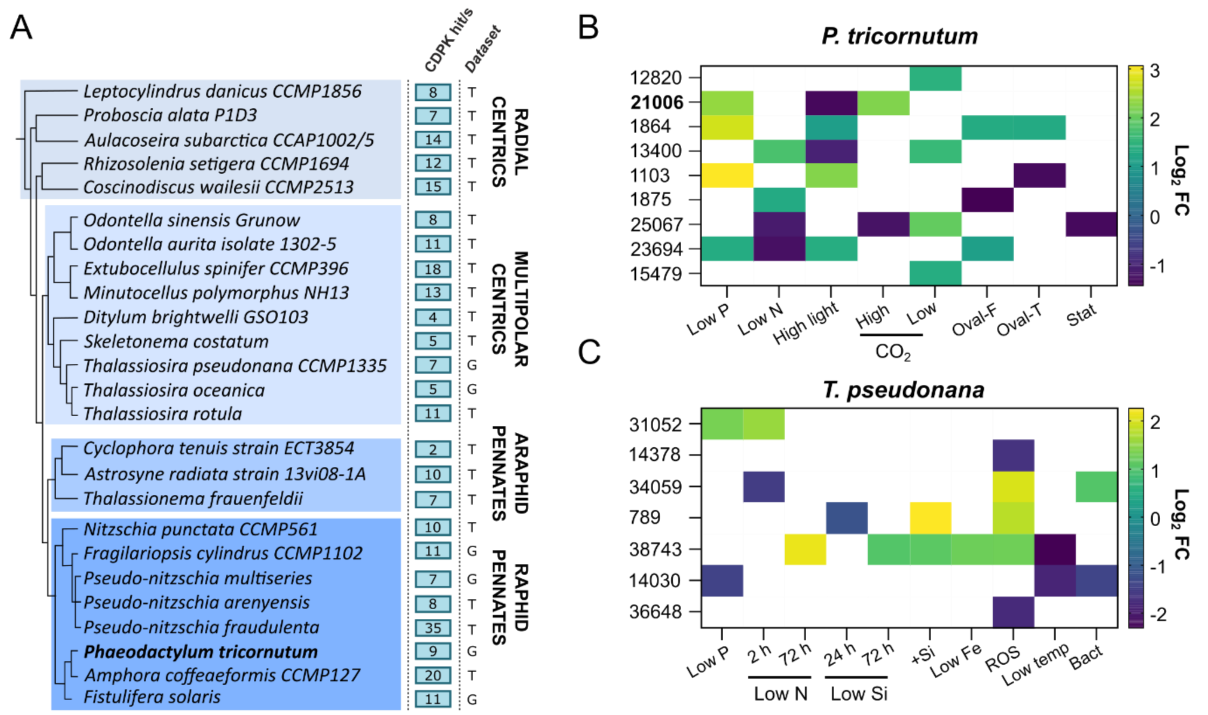

Figure S5. CDPKs are abundant and widely distributed across pennate and centric diatoms. A. The number of CDPKs identified in sequenced centric and pennate diatoms, using PtCDPK2 (protein id. 21006) as a query sequence. Data are from transcriptome (T) and genome (G) databases (**Materials and Methods**). An e-value cut off score $1E^{-25}$ was used, and all hits carefully scrutinised for the presence of both a kinase and EF hand domain/s. Note, results derived from transcriptional datasets may miss genes not expressed during RNA harvesting. **B**. Transcriptional expression of *P. tricornutum* CDPK hits in response to different environmental stressors using the DiatOmicBase portal (Villar et al. 2025). Data is displayed for transcripts showing significantly differential expression (e-value<0.05 with a $\log_2$ fold change (FC) less than -1 or greater than 1). Data is compiled from experimental studies as follows: low P (P deplete versus replete control) (Li et al. 2022a), low N (N replete versus N deplete) (Levitan et al. 2015), high light (versus control after 24h) (Kan et al. 2023), high CO_2 (Zhang et al. 2020) and low CO_2 (Levering et al. 2017), oval versus fusiform (Oval-T) and versus triradiate morphotype (Oval-T) (Ovide et al. 2018), as well as stationary (Stat) versus early growth culture phase (Kwon et al. 2021). **C**. As in B, but for CDPK hits identified in the centric diatom *T. pseudonana*. Data is compiled from experimental studies as follows: Low P (P deplete versus replete control after 72 h), low (N deplete versus replete control after 2 h and 72 h), low si (silica starvation after 24 h and 72 h) and 4 h following resupply (+Si), low Fe (iron starvation versus control after 72 h), are all from (Li et al. 2022b). H_2O_2 treatment versus control from (Graff van Creveld et al. 2023), low temperature 6 h versus control (Li et al. 2022b) and following exposure to bacterial filtrate (Bact) from (Bartolek et al. 2022).

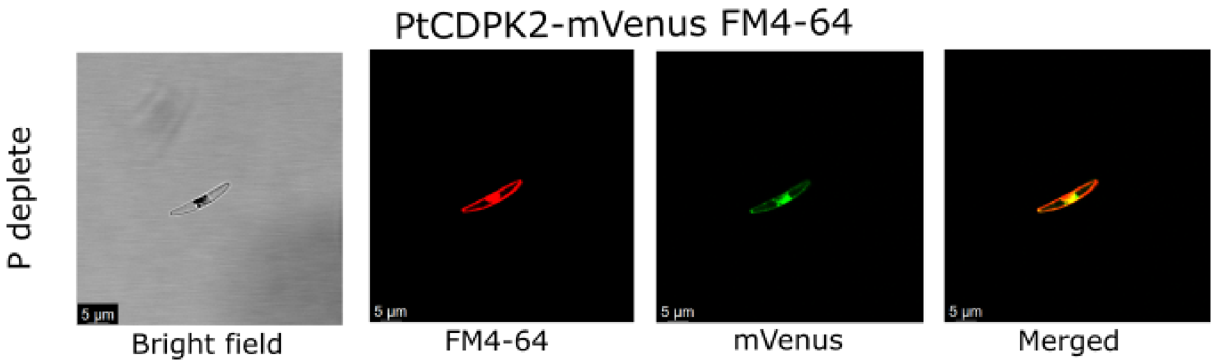

Figure S6. PtCDPK2-mVenus expression and localisation under P-deplete conditions. Localisation of PtCDPK2-mVenus and plasma membrane stain FM4-64 of 4-day old cells grown in low phosphate (1.8 µM) medium and imaged using confocal microscopy.

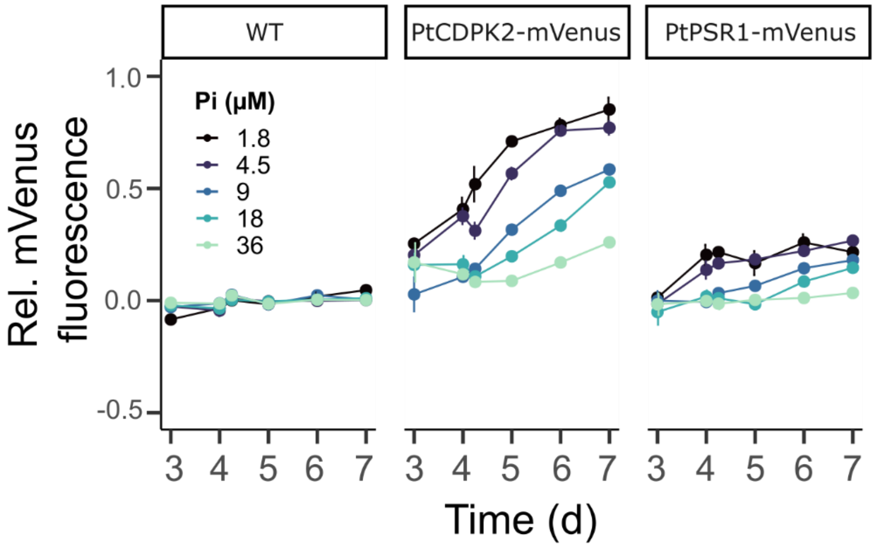

Figure S7. PtCDPK2-mVenus and PtPSR1-mVenus are upregulated under P-starvation. Relative mVenus fluorescence (mVenus/chlorophyll fluorescence) of wild-type (WT), PtCDPK2-mVenus and PtPSR1-mVenus strains over 7 days inoculated into different initial concentrations of phosphate (Pi). Data are presented as mean ± standard error (n=3).

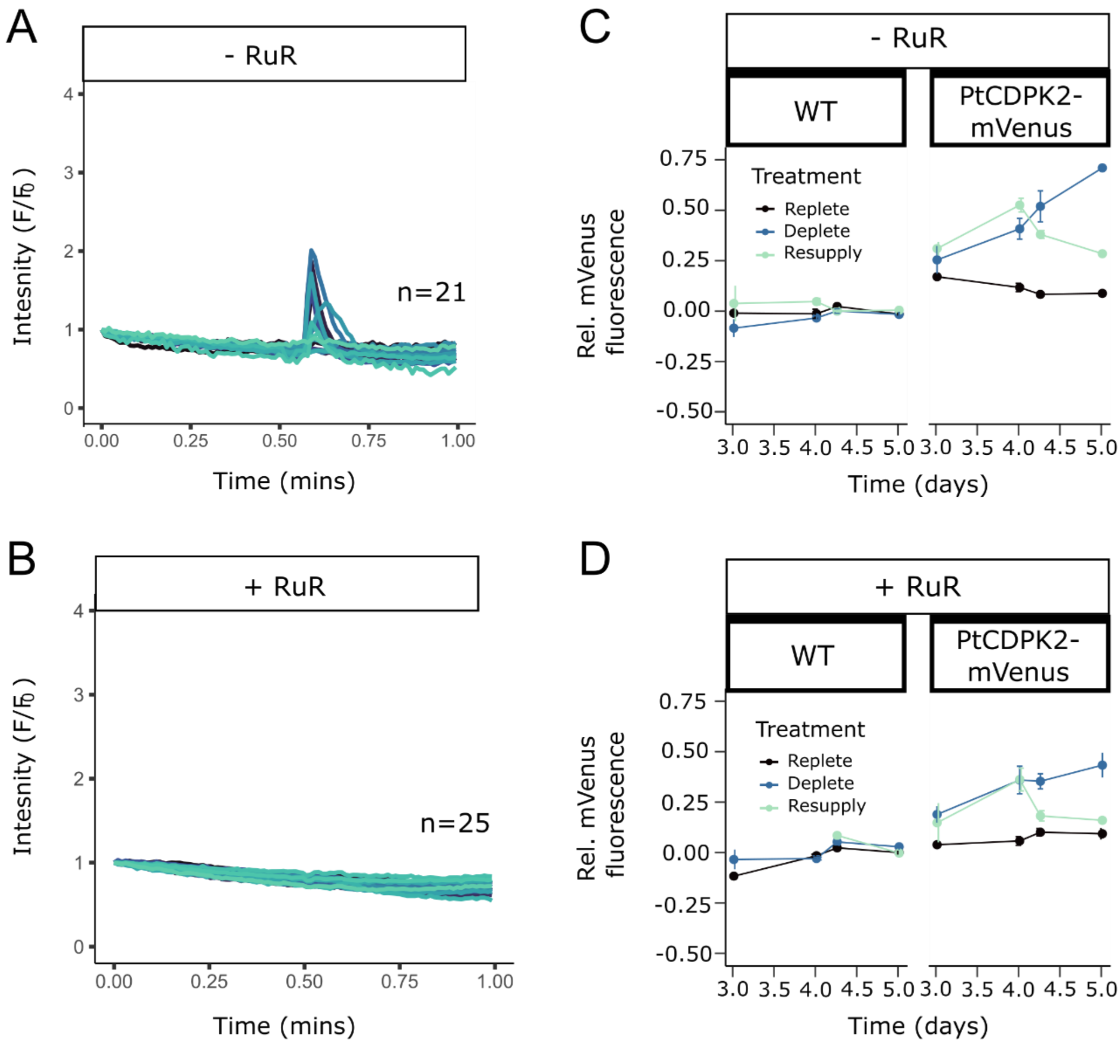

Figure S8. Decreases in PtCDPK2-mVenus fluorescence following phosphate resupply to P deplete cells are not dependent on P-Ca^{2+} signalling. A) Fluorescence traces (F/F_0) of 4-day old R-GECO1 (PtR1) cells grown in P deplete (1.8 µM phosphate) conditions exposed to 36 µM phosphate resupply. Cells without RuR were perfused with filtered seawater (FSW) for 30 s and then FSW with 36 µM phosphate for 30 s. **B**) Experiment as described in (A) except cells were pre-treated with 5 µM ruthenium red (RuR) for 5 mins. **C**) PtCDPK2-mVenus and WT mVenus/chlorophyll fluorescence in replete (36 µM phosphate), deplete (1.8 µM phosphate) and resupply (1.8 µM day 4 cells resupplied with 36 µM Pi) without RuR. **D**) As in C, except RuR was applied to all treatments on day 4, just prior to phosphate resupply in the resupply treatment. Data in C and D are mean ± standard error (n=3).

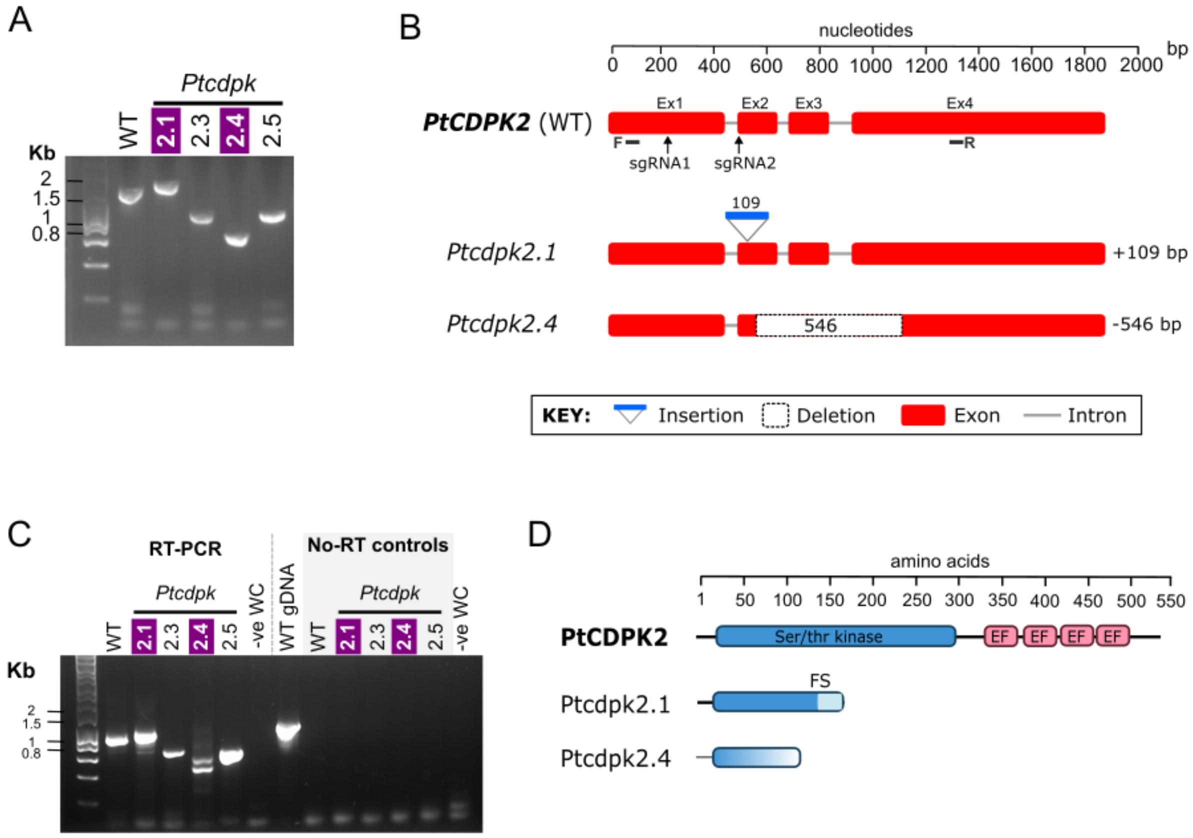

Figure S9. Screening *Ptcdpk2* mutants. A) PCR screening of wildtype (WT) and putative *Ptcdpk2* knockout lines using primers Keh 409F (F) and Keh 410R (R). **B)** Nucleotide sequence of WT *PtCDPK2* and mutants *Ptcdpk2.1* and *Ptcdpk2.4* (Supplementary Information 3-4). Exons are red, and grey lines are introns. Black arrows indicate single guide RNA (sgRNA) locations. Primer binding sites for Keh 409F (F) and Keh 410R (R) are indicated. Sizes of indels are indicated to the right, as confirmed through Sanger sequencing. Sequencing data revealed no evidence of ambiguous nucleotide sequences, indicating identical indels on both alleles. **C)** PCR screening of cDNA from WT and *Ptcdpk2* mutants via reverse transcriptase (RT)-PCR. Negative no template water controls (-ve WC), and no-RT controls using RNA samples but with *Taq* polymerase, without RT are indicated. No-RT controls were further validated using genomic DNA extracted from WT *P. tricornutum* (WT gDNA). **D)** Predicted protein structure of *Ptcdpk2.1* and *Ptcdpk2.4* compared to WT, deduced from the RT-PCR analysis, indicating positions of frameshifts (FS) and truncations.

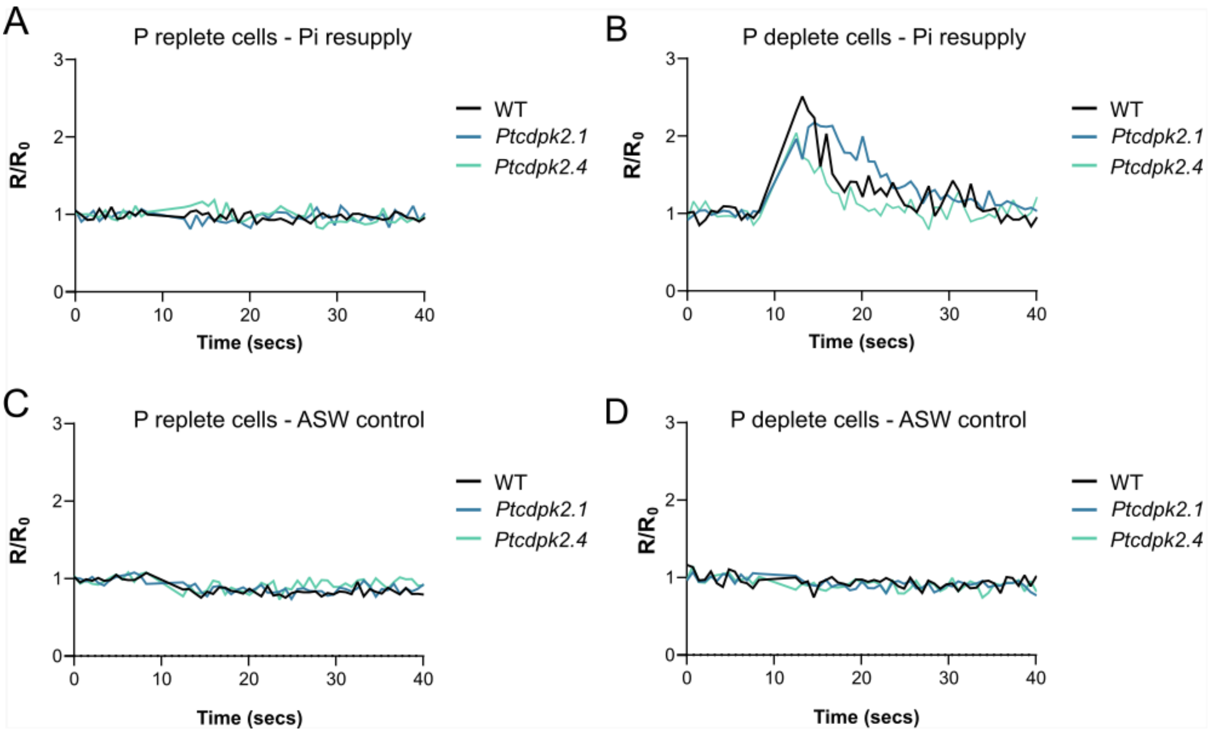

Figure S10. Examining the capacity of *Ptcdpk2* mutants for phosphate-induced Ca^{2+} signalling. Example Ca^{2+} signalling trace graphs for data shown in Figure 5C for: **A)** WT, **B)** *Ptcdpk2.1* and **C)** *Ptcdpk2.4* expressing Ca^{2+} indicator R-GECO1-mTurquoise (RGMT). R_0 is the R value at the start of the experiment (I.e. time 0) and R is the ratio of R-GECO1/mTurquoise fluorescence. Cultures were grown in phosphate replete (36 µM phosphate) or deplete (1.8 µM phosphate) conditions for 4 days, and then resupplied with 36 µM phosphate after ~8 seconds. The experiment was repeated on four independent occasions (each with two technical replicates) per line, with similar results. Control experiments showing traces of P replete and deplete cells injected with artificial seawater (ASW) rather than ASW + phosphate are shown in C and D, respectively.
